# An MRI-based model for preoperative prediction of tertiary lymphoid structures in patients with gallbladder cancer

**DOI:** 10.1186/s13244-025-02007-4

**Published:** 2025-08-30

**Authors:** Ying Xu, Zhuo Li, Weihua Zhi, Yi Yang, Jingzhong Ouyang, Yanzhao Zhou, Zeliang Ma, Sicong Wang, Lizhi Xie, Jianming Ying, Jinxue Zhou, Xinming Zhao, Feng Ye

**Affiliations:** 1 Department of Diagnostic Radiology, National Cancer Center/National Clinical Research Center for Cancer/Cancer Hospital, Chinese Academy of Medical Sciences and Peking Union Medical College, Beijing, China; 2 Department of Pathology, National Cancer Center/National Clinical Research Center for Cancer/Cancer Hospital, Chinese Academy of Medical Sciences and Peking Union Medical College, Beijing, China; 3 State Key Laboratory of Molecular Oncology, National Cancer Center/National Clinical Research Center for Cancer/Cancer Hospital, Chinese Academy of Medical Sciences and Peking Union Medical College, Beijing, China; 4 Department of Hepatobiliary Surgery, National Cancer Center/National Clinical Research Center for Cancer/Cancer Hospital, Chinese Academy of Medical Sciences and Peking Union Medical College, Beijing, China; 5 Beijing Key Laboratory of Cell and Gene Therapy for Digestive System Tumor, Beijing, China; 6 Key Laboratory of Gene Editing Screening and Research and Development (R&D) of Digestive System Tumor Drugs, Chinese Academy of Medical Sciences and Peking Union Medical College, Beijing, China; 7 Department of Hepatobiliary and Pancreatic Surgery, The Affiliated Cancer Hospital of Zhengzhou University & Henan Cancer Hospital, Zhengzhou, China; 8 Department of Medical Oncology, The Affiliated Cancer Hospital of Zhengzhou University & Henan Cancer Hospital, Zhengzhou, China; 9 University of Electronic Science and Technology of China, Chengdu, China; 10 Department of Radiation Oncology, National Cancer Center/National Clinical Research Center for Cancer/Cancer Hospital, Chinese Academy of Medical Sciences and Peking Union Medical College, Beijing, China; 11 Magnetic Resonance Imaging Research, General Electric Healthcare, Beijing, China

**Keywords:** Magnetic resonance imaging, Radiomics, Tertiary lymphoid structure, Gallbladder cancer

## Abstract

**Objectives:**

To predict tertiary lymphoid structures (TLSs) in gallbladder cancer (GBC) using preoperative magnetic resonance imaging (MRI)-based radiomics.

**Methods:**

Patients with GBC from two centres served as training (*n* = 129) and external validation (*n* = 44) cohorts. Radiomics features were extracted from six imaging sequences for inclusion in a radiomics model (Rad-score). Univariate and multivariate logistic regression were used to identify independent clinico-radiological predictors of TLS status. The clinical and radiomics models were integrated into a combined model. Areas under receiver operating characteristic curves (AUC) were used to assess model performance. The combined model was divided into low- and high-risk according to the cut-off value determined by the maximum Youden index of the ROC.

**Results:**

Intratumoural TLSs independently predicted RFS (*p* = 0.046). Eight features were included in the Rad-score. The clinical model included three independent predictors of TLS status (tumour height, liver invasion, and arterial-phase hypo-enhancement). In the training cohort, the combined model outperformed the separate clinical and radiomics models (AUC, 0.891 vs 0.870 and 0.775, respectively) and was externally valid. In both training and external cohorts, RFS in the low-risk group was substantially higher compared to the high-risk group. The low-risk group in the immunotherapy cohort had a significantly higher median overall survival than the high-risk group.

**Conclusions:**

The MRI-based combined model developed in this study can preoperatively predict intratumoural TLS status. It accurately stratified the RFS of patients after surgery and the OS of patients with immunotherapy.

**Critical relevance statement:**

This combined model is useful for predicting response and prognosis, not only for the recurrence-free survival of patients with GBC who have undergone surgery, but also for the overall survival of patients who have received immunotherapy

**Key Points:**

- Intratumoural TLSs independently predict recurrence-free survival of GBC.
- Our MRI-based combined model is a preoperative TLS marker.
- The combined model accurately stratifies postoperative/post-immunotherapy recurrence-free and overall survival of GBC.

**Graphical Abstract:**

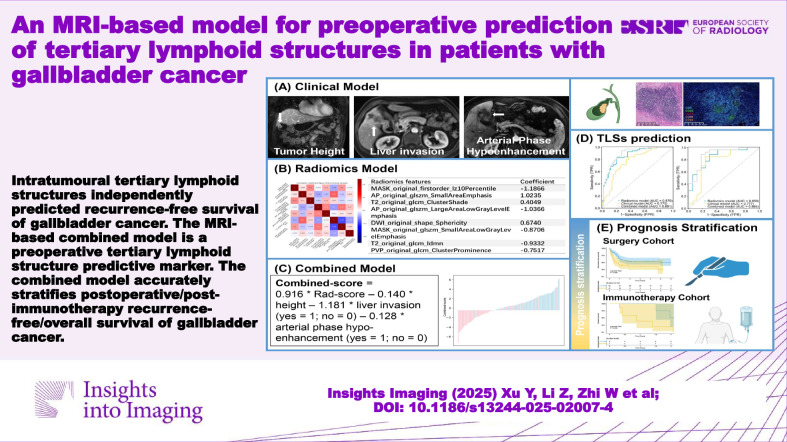

## Introduction

Biliary tract cancers (BTCs) include cholangiocarcinoma (intrahepatic, perihilar and extrahepatic) and gallbladder cancer (GBC), with GBC being the most prevalent type [1, 2]. GBC is characterised by aggressiveness and poor prognosis. Surgical resection is the only treatment with curative intent for GBC, but it is unsuitable in 70–90% of cases due to diagnosis at advanced stages [3, 4]. Immunotherapy-based combination therapy has become the first-line treatment for unresectable and metastatic GBC [5, 6].

Accurate efficacy and prognostic markers are particularly important indicators for patients undergoing immunotherapy. Currently, indicators derived from laboratory tests (e.g., nutritional parameters, tumour markers, and gene expression signatures) provide the basis for the assessment of GBC prognoses [7]. The 2023 clinical guidelines of the National Comprehensive Cancer Network recommend testing for microsatellite instability, mismatch repair and/or tumour mutational burden in cases of unresectable or metastatic GBC [5]. However, genetic testing is expensive and time-consuming, and does not allow for the intuitive analysis of entire tumours. Tumour-infiltrating CD8^+^ lymphocyte counts and programmed death ligand-1 (PD-L1) expression have been linked to GBC patient survival, but their prognostic value remains controversial and needs to be explored [8–10].

Recent research has focused on tumour-associated tertiary lymphoid structures (TLSs), which are ectopic immune-cell aggregates similar to those in secondary lymphoid organs [11]. The presence of these structures has potential prognostic value and is associated with improved immunotherapy responses for intrahepatic cholangiocarcinoma (ICC) and other solid tumours (e.g., pancreatic ductal, lung adenocarcinomas and hepatocellular carcinoma) [12–19].

Ding et al [20] found that the presence of TLSs in the intratumoural region in ICC correlated positively with favourable prognoses, and established a subregional quaternary scoring system according to TLS abundance (with 0 representing TLS-negative ICC and 1–3 representing TLS-positive ICC). GBC in a certain location (neck) mimics hilar or mid-common bile duct cholangiocarcinoma [4], but no association of TLSs with the prognosis of GBC has been reported.

Additionally, TLSs can be assessed only postoperatively. A non-invasive means for preoperative TLS evaluation in patients with GBC is needed. In this context, magnetic resonance imaging (MRI) is a critical tool for differential diagnosis, preoperative staging, prognostic stratification and the assessment of treatment response [1, 21–23]. Radiomics analysis enables the quantification of parameters reflecting tumour pathophysiology, phenotypes and internal heterogeneity that cannot be assessed via imaging visualisation, thereby improving clinical decision making [24, 25].

This study was conducted to explore the prognostic value of TLSs for GBC and to develop a non-invasive MRI-based radiomics model for the preoperative prediction of the intratumoural TLS status. The performance of the model in prognostic stratification for immunotherapy was tested with a cohort of patients with unresectable GBC, details of the cohort were registered on ClinicalTrials.gov (NCT03996408) [26].

## Methods

### Patient selection

**Fig. 1:**
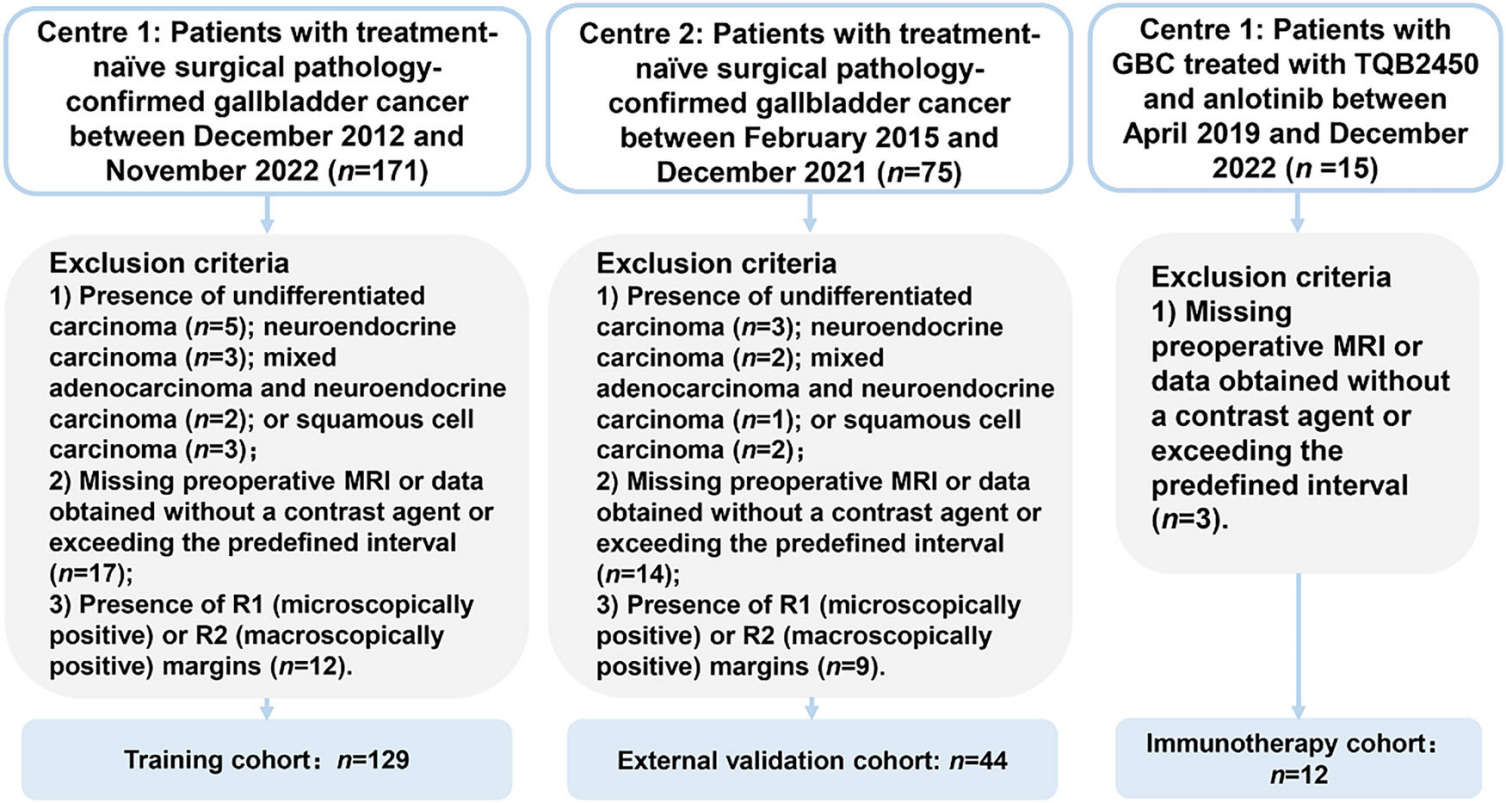
The flowchart of the patient selection process

Due to the retrospective nature of the analysis, the informed consent requirement was waived by the institutional review boards of the participating hospitals, which approved the study (no. 19/164-1948). We reviewed the records of patients with GBC treated at centre 1 (*n* = 171) between December 2012 and November 2022 and centre 2 (*n* = 75) between February 2015 and December 2021. The inclusion criteria were: (1) presence of treatment-naïve, surgical pathology–confirmed GBC and (2) availability of preoperative (≤ 1 month before surgery) data from multiphase contrast-enhanced MRI examination of the liver. The exclusion criteria were: (1) presence of undifferentiated carcinoma, neuroendocrine carcinoma, mixed adenocarcinoma and neuroendocrine carcinoma, or squamous cell carcinoma; (2) missing preoperative liver MRI data, or data obtained without contrast agent use or outside of the predefined interval; (3) previous receipt of treatment for gallbladder lesions; and (4) presence of R1 (microscopically positive) or R2 (macroscopically positive) margins. The application of these criteria led to the inclusion of centre 1 (*n* = 129) in the training cohort and centre 2 (*n* = 44) in the external validation cohort. An immunotherapy cohort (*n* = 12) of patients with advanced GBC who refused or were ineligible for first-line chemotherapy, or whose GBC progressed after this treatment, was selected from a phase-Ib clinical trial (NCT03825705) [26] and the real world. Figure 1 is a flowchart showing the patient choice procedure.

### Clinical and radiological evaluation

Clinical data on patients’ demographic characteristics and preoperative tumour markers (cancer embryonic antigen and carbohydrate antigen 19-9) were extracted from medical records. Two radiologists (Y.X. and F.Y.) with 6 years and 20 years of experience in abdominal radiology, respectively, who were blinded to clinical and pathological findings, independently assessed images from preoperative multiparametric MRI examinations (sequence details are presented in Supplementary Material 1: Table 1). They evaluated the lesion parameters listed in Supplementary Material 2: Table 2. Quantitative enhancement values (the contrast enhancement ratio and lesion-to-liver and lesion-to-muscle contrast ratios) for the arterial, portal venous and delayed phases are presented in Supplementary Material 3. Inter-observer agreement was assessed using interclass correlation coefficients, and only variables with coefficients > 0.80 were retained for inclusion in the analysis.

### Histopathological evaluation

**Fig. 2:**
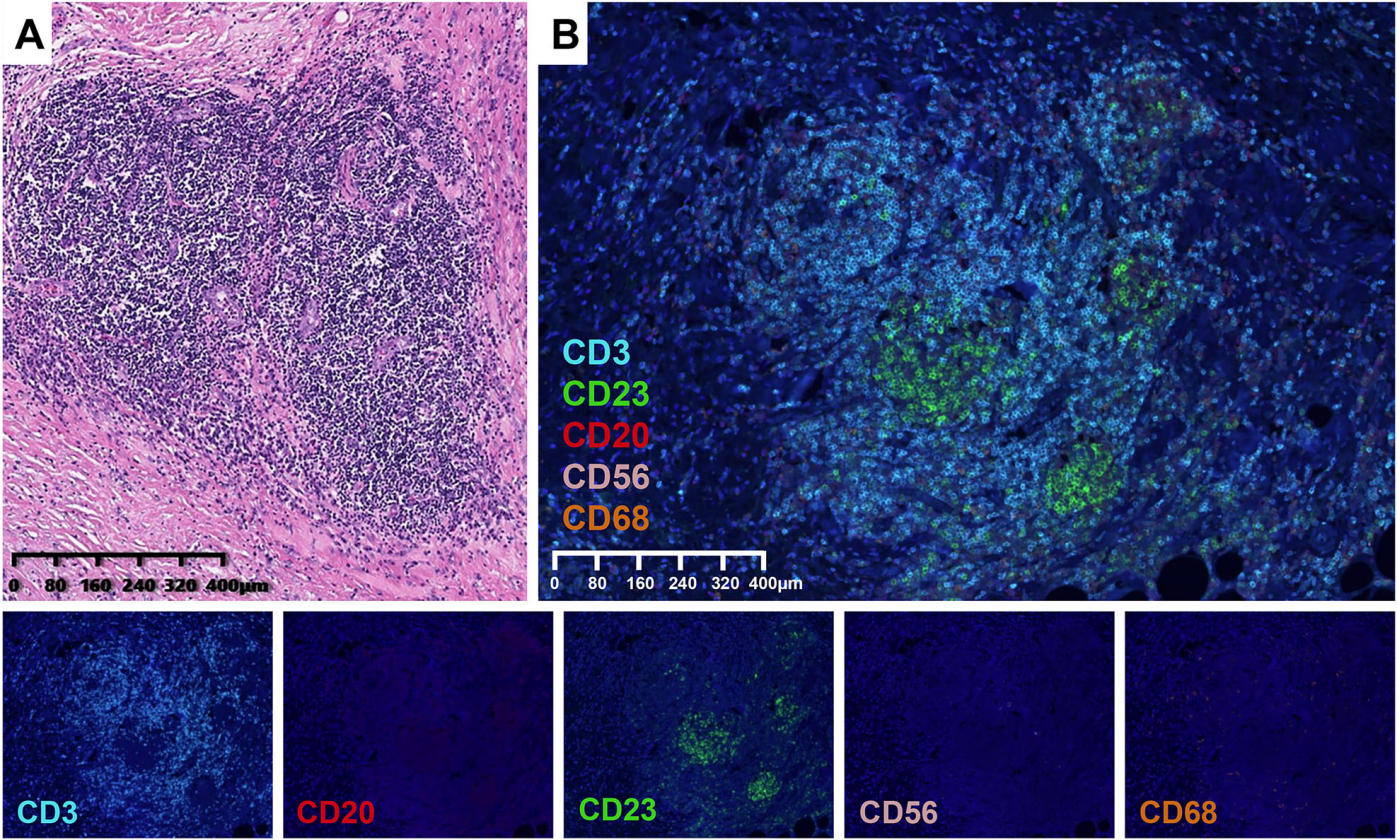
Histological identification of intratumoural TLSs in GBC. H&E staining shows mature TLS with germinal centre formation and endothelial distribution (**A**). Multiple immunofluorescence detection proved complex of cell types for the immune microenvironment of GBC (**B**). Please note the abundant B lymphocytes (CD20 labelled), T lymphocytes (CD3 labelled), NK cells (CD56 labelled), macrophages (CD68 labelled) and most importantly, CD23 positive cells in the follicular centre, the proof for mature TLS (magnification for 40× in all)

A pathologist (unaware of clinical and radiologic results) with 10 years of oncological experience (Z.L.) reviewed images of whole haematein-eosin saffron–stained slides of the GBC lesions, and a second blinded pathologist with 20 years of oncological experience (J.Y.) confirmed the findings. Discrepancies were discussed until consensus had been reached. The pathologists morphologically classified intratumoural TLSs according to their maturity as (1) lymphoid aggregates (poorly-defined lymphocyte clusters), (2) primary lymphoid follicles lacking germinal centres, and (3) secondary lymphoid follicles with germinal centres (Fol-IIs), as described previously [20, 27, 28]. Tumours lacking Foll-IIs were defined as intratumoural TLS negative, and those with one or more Fol-IIs were defined as intratumoural TLS positive. An example of the histological identification of intratumoural TLSs in GBC lesions is shown in Figs. 2.

### Radiomics analysis

The radiomics procedure consisted of manual tumour segmentation, feature extraction and selection, multiple sequence fusion, volume-of-interest (VOI) fusion and radiomics score (Rad-score) calculation. On axial non-enhanced T1-weighted, T2-weighted, diffusion-weighted, arterial phase, portal venous phase and delayed-phase images, VOIs within the visible tumour borders were delineated manually using ITK-SNAP by Radiologist 1 (Y.X.) [29]. The examples of tumour segmentation for three tumour morphological types (wall thickening, polypoid and mass forming) were presented as Supplementary Material 4: Figs. 1–3. The tumour resegmentation was performed by a second independent radiologist (Radiologist 2: F.Y.) for images of 30 patients selected randomly, and features with inter-observer agreement of more than 0.80 were reserved. After image pre-processing, 642 features per patient were extracted from these imaging sequences (Supplementary Material 5). Redundant features were eliminated through variance, and irrelevant features were discarded through correlation, reducing the count to 78. Subsequently, multivariate logistic regression analysis was employed to select a final set of features for the radiomics model (Rad-score).

### Patient follow-up

During the first two years following surgery, the patients were monitored every three months. From the third to the fifth year, the patients were monitored every six months, and then once a year after that. GBC recurrence was evaluated using MRI, computed tomography (CT) or positron emission tomography. Recurrence-free survival (RFS) was evaluated as the interval from surgery to cancer recurrence or final follow-up. Overall survival (OS) was evaluated as the interval from surgery to any-cause death or final follow-up. The last follow-up examination occurred on 10 June 2023.

### Immunotherapy regimen

This immunotherapy cohort collected patients from the Phase Ib Clinical Study on Safety and Efficacy of TQB2450, injection Combined With Anlotinib Hydrochloride Capsule in Patients With Advanced Biliary Adenocarcinoma [26], which is mainly aimed at patients with biliary tract tumours who had previously failed first-line chemotherapy. In the real-world setting, we also adopted the same regimens. Twelve GBC patients with complete imaging data were selected from our centre. Details of the GBC immunotherapy cohort were registered on ClinicalTrials.gov (NCT03825705).

### Statistical analysis

To identify clinical factors linked to RFS, univariate Cox regression analysis was used, and variables (*p* < 0.05) were added to the multivariate Cox regression model. The same method was applied to univariate and multivariate logistic regression in order to assess the relationships between clinical and radiological factors and TLSs. For each variable, relative risk was represented using odds ratios (ORs) with 95% confidence intervals (CIs). Areas under the curve (AUCs) and receiver operating characteristic curves (ROCs) were used to assess the clinical model’s and Rad-score’s discriminatory performance. To compare AUCs, the DeLong test was employed. Corresponding sensitivity, specificity and accuracy values were also compared. The maximum Youden index of the ROC was used to calculate the combined model cut-off value. Calibration curves and the Hosmer–Lemeshow test were used to assess consistency between predicted and observed values. Decision curve analysis (DCA) was used to assess the clinical utility of the combined model. Survival according to TLS status and combined model estimates was examined using the Kaplan–Meier method and log-rank test. The statistical analyses were performed using SPSS (version 25.0; IBM), R statistical software (version 3.3.3), and Python (version 3.5.6). *p* < 0.05 was used as the significance level.

## Results

### Baseline characteristics

The training and external validation cohorts consisted of 129 (45.7% male) and 44 (45.5% male) patients with GBC, respectively, with a median age of 62 [interquartile ranges (IQRs), 58–67 and 56–66, respectively] years. No significant difference in any clinical, radiological, or pathological variable was observed between the training and external validation cohorts, ensuring their comparability (Table 1).

**Table 1:** Baseline clinical, radiological, and pathologic characteristics of patients with GBC in the training and external validation cohorts

Characteristics	Training cohort	External validation cohort	*p* value
*n*	129	44	
Preoperative variables			
Male, *n* (%)	59 (45.7%)	20 (45.5%)	0.974
Age, median (IQR)	62 (58, 67)^a^	62 (56, 66)^a^	0.528
HBV-positive, *n* (%)	47 (36.4%)	13 (29.5%)	0.407
HCV-positive, *n* (%)	3 (2.3%)	2 (4.5%)	0.812
Cholecystitis, *n* (%)	7 (5.4%)	2 (4.5%)	1.000
Gallstone, *n* (%)	25 (19.4%)	9 (20.5%)	0.877
Liver cirrhosis, *n* (%)	5 (3.9%)	2 (4.5%)	1.000
Fatty liver, *n* (%)	13 (10.1%)	6 (13.6%)	0.709
CA199 > 37 U/mL, *n* (%)	55 (42.6%)	17 (38.6%)	0.642
CEA > 5 ng/mL, *n* (%)	39 (30.2%)	14 (31.8%)	0.844
Location, *n* (%)			
Neck	26 (20.2%)	8 (18.2%)	0.776
Body	27 (20.9%)	12 (27.3%)	0.385
Fundus	51 (39.5%)	15 (34.1%)	0.521
Diffuse	25 (19.4%)	9 (20.5%)	0.877
Size, median (IQR)			
Length	3.4 (2.3, 4.8)^a^	3.35 (2.275, 4.525)^a^	0.522
Width	2.1 (1.6, 3.5)^a^	2.55 (1.675, 3.525)^a^	0.521
Height	2.6 (1.8, 4.1)^a^	2.7 (1.9, 4.125)^a^	0.911
Tumour type, *n* (%)			
Wall thickening	66 (51.2%)	21 (47.7%)	0.694
Polypoid	35 (27.1%)	13 (29.5%)	0.757
Mass forming	28 (21.7%)	10 (22.7%)	0.888
Involved wall, *n* (%)			
Hepatic side	9 (7%)	1 (2.3%)	0.435
Peritoneal side	5 (3.9%)	2 (4.5%)	1.000
Both sides	115 (89.1%)	41 (93.2%)	0.629
Liver invasion, *n* (%)	64 (49.6%)	23 (52.3%)	0.761
Bile duct invasion, *n* (%)	24 (18.6%)	6 (13.6%)	0.452
Suspicious lymph nodes (imaging), *n* (%)	65 (50.4%)	24 (54.5%)	0.634
Liver metastasis, *n* (%)	6 (4.7%)	1 (2.3%)	0.804
T stage (imaging), *n* (%)			0.941
I	7 (5.4%)	2 (4.5%)	
II	61 (47.3%)	19 (43.2%)	
III	59 (45.7%)	22 (50%)	
IV	2 (1.6%)	1 (2.3%)	
Intrahepatic bile duct dilatation, *n* (%)	54 (41.9%)	14 (31.8%)	0.239
Extrahepatic bile duct dilatation, *n* (%)	58 (45%)	15 (34.1%)	0.207
T1WI signal, *n* (%)			0.833
Iso- to hyperintense	61 (47.3%)	20 (45.5%)	
Hypointense	68 (52.7%)	24 (54.5%)	
T2WI signal, *n* (%)			1.000
Hyperintense	125 (96.9%)	43 (97.7%)	
Iso- to hypointense	4 (3.1%)	1 (2.3%)	
DWI signal, *n* (%)			0.928
hyperintense	87 (67.4%)	30 (68.2%)	
slightly hyperintense	42 (32.6%)	14 (31.8%)	
Arterial phase enhancement, *n* (%)			
Hyperenhancement	64 (49.6%)	21 (47.7%)	0.829
Ring enhancement	42 (32.6%)	18 (40.9%)	0.315
Hypoenhancement	23 (17.8%)	5 (11.4%)	0.315
Delayed enhancement, *n* (%)	19 (14.7%)	7 (15.9%)	0.850
Wash in and wash out, *n* (%)	31 (24%)	9 (20.5%)	0.627
Continuous enhancement, *n* (%)	79 (61.3%)	28 (63.6%)	0.778
Arterial phase quantification, mean ± SD			
CER_AP_	1.82 ± 1.26^b^	1.70 ± 1.21^b^	0.570
LLC_AP_	0.42 ± 0.55^b^	0.45 ± 0.57^b^	0.754
LMC_AP_	0.72 ± 0.83^b^	0.86 ± 0.84^b^	0.353
Portal vein phase quantification, mean ± SD			
CER_PVP_	2.23 ± 1.18^b^	2.15 ± 1.18^b^	0.720
LLC_PVP_	0.11 ± 0.43^b^	0.08 ± 0.44^b^	0.756
LMC_PVP_	0.68 ± 0.89^b^	0.78 ± 0.89^b^	0.522
Delayed phase quantification, mean ± SD			
CER_DP_	1.91 ± 1.25^b^	2.06 ± 1.45^b^	0.503
LLC_DP_	0.15 ± 0.33^b^	0.18 ± 0.32^b^	0.587
LMC_DP_	1.01 ± 1.01^b^	0.98 ± 0.88^b^	0.890
Necrosis, *n* (%)	29 (22.5%)	9 (20.5%)	0.779
Intratumoural vessels, *n* (%)	9 (7%)	2 (4.5%)	0.831
Peripheral liver parenchyma enhancement, *n* (%)	72 (55.8%)	23 (52.3%)	0.684
Postoperative variables			
Intratumoural TLSs, *n* (%)	70 (54.3%)	27 (61.4%)	0.413
Surgical method, *n* (%)			0.757
Simple cholecystectomy	16 (12.4%)	7 (15.9%)	
Extended cholecystectomy	22 (17.1%)	5 (11.4%)	
Extended cholecystectomy plus hepatic lobectomy	87 (67.4%)	30 (68.2%)	
Whipple or pancreaticoduodenectomy	4 (3.1%)	2 (4.5%)	
Differentiation, *n* (%)			0.421
High	9 (7%)	1 (2.3%)	
Moderate	44 (34.1%)	13 (30.2%)	
Low	76 (58.9%)	29 (67.4%)	
AJCC 8th T stage (pathology), *n* (%)			0.965
I	15 (11.6%)	4 (9.1%)	
II	48 (37.2%)	16 (36.4%)	
III	58 (45%)	21 (47.7%)	
IV	8 (6.2%)	3 (6.8%)	
N stage (pathology), *n* (%)			0.793
N0	75 (58.1%)	23 (52.3%)	
N1	44 (34.1%)	17 (38.6%)	
N2	10 (7.8%)	4 (9.1%)	
Liver invasion, *n* (%)	49 (38%)	18 (40.9%)	0.731
Nerve invasion, *n* (%)	58 (45%)	21 (47.7%)	0.750
Blood vessel invasion, *n* (%)	49 (38%)	19 (43.2%)	0.542
Adjuvant therapy, *n* (%)	53 (41.1%)	19 (43.2%)	0.808

Data are the number of patients unless otherwise indicated

Student’s *t*-test or Mann–Whitney *U*-test were used for the continuous data. Categorical data are compared by using the χ^2^-test or the Fisher exact test, as much as possible

*IQR* interquartile range, *HBV* hepatitis B virus, *HCV* hepatitis C virus, *CA199* carbohydrate antigen 199, *CEA* carcinoembryonic antigen, *WI* weighted imaging, *DWI* diffusion weighted imaging, *CER* contrast enhancement ratio = (AP_mean_ − Mask_mean_)/Mask_mean_ or (PVP_mean_ − Mask_mean_)/Mask_mean_ or (DP_mean_ − Mask_mean_)/Mask_mean_, *LLC* lesion-to-liver contrast ratio = (AP_mean_ − AP_live_)/AP_liver_ or (PVP_mean_ − PVP_live_)/PVP_liver_ or (DP_mean_ − DP_live_)/DP_liver_, *LMC* lesion-to-muscle contrast ratio = (AP_mean_ − AP_muscle_)/AP_muscle_ or (PVP_mean_ − PVP_muscle_)/PVP_muscle_ or (DP_mean_ – DP_muscle_)/DP_muscle_, *TLSs* tertiary lymphoid structures, *AJCC* American Joint Committee on Cancer

^a^ Data are median (inter-quartile range)

^b^ Data are means ± standard deviation

^*^ Data are statistically significant results

### Association of TLSs with RFS

**Fig. 3:**
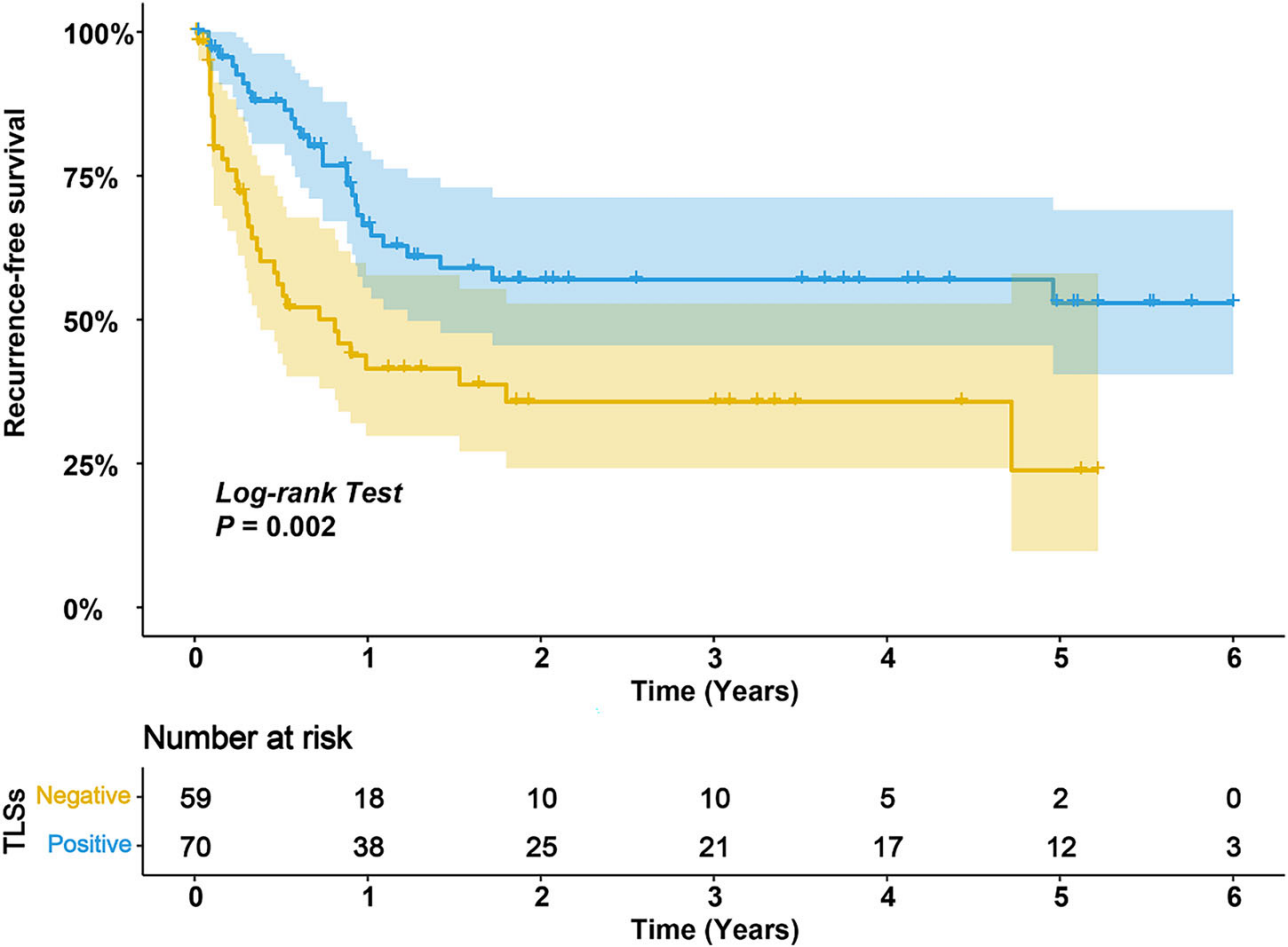
Kaplan–Meier curves for RFS of 129 patients with GBC as categorised by the TLS status in centre 1

The univariate Cox regression analysis yielded nine clinical characteristics (included in the multivariate model) associated with RFS in the training cohort (*p* < 0.05). The presence of intratumoural TLSs was the only independent predictive factor for RFS in the multivariate analysis (hazard ratio,1.86; 95% CI: 1.01–3.43; *p* = 0.046; Supplementary Material 6: Table 3). In the training cohort, the RFS of patients with intratumoural TLSs was significantly longer than that of those without intratumoural TLSs (*p* = 0.002; Fig. 3).

### Clinical model

One variable with a low interclass correlation coefficient (intratumoural vessels, 0.69; 95% CI: 0.59–0.77) was excluded from the clinical model (Supplementary Material 7: Table 4). Of 19 training-cohort variables entered into the multivariate logistic regression analysis (univariate *p* < 0.05), three variables (tumour height: OR 0.67, 95% CI: 0.52–0.86, *p* = 0.002; liver invasion: OR 0.37, 95% CI: 0.16–0.84, *p* = 0.02; arterial phase hypo-enhancement: OR 0.33, 95% CI: 0.11–1.00, *p* = 0.0496) were found to independently predict TLS status (Table 2).

**Table 2:** Univariate and multivariate logistic regression analysis of clinical, radiological characteristics of patients with GBC for TLSs in the training cohort

Characteristics	Total (*N*)	Univariate analysis		Multivariate analysis	
		Odds ratio (95% CI)	*p* value	Odds ratio (95% CI)	*p* value
Sex	129				
Male	59	Reference			
Female	70	1.458 (0.725–2.935)	0.290		
Age	129	0.962 (0.925–1.001)	0.058		
Cholecystitis	129				
Yes	122	Reference			
No	7	1.624 (0.349–7.567)	0.537		
Gallstone	129				
Yes	25	1.119 (0.467–2.684)	0.800		
No	104	Reference			
CA199 > 37 U/mL	129				
Yes	55	1.114 (0.553–2.244)	0.763		
No	74	Reference			
CEA > 5 ng/mL	129				
Yes	39	2.156 (1.004–4.633)	0.049^a^		
No	90	Reference			
Location, neck	129				
Yes	26	0.561 (0.229–1.374)	0.206		
No	103	Reference			
Location, body	129				
Yes	27	0.520 (0.214–1.265)	0.150		
No	102	Reference			
Location, fundus	129				
Yes	51	Reference			
No	78	1.190 (0.585–2.420)	0.632		
Location, diffuse	129				
Yes	25	5.067 (1.865–13.762)	0.001^a^		
No	104	Reference			
Size, length	129	1.619 (1.286–2.038)	< 0.001^a^		
Size, width	129	1.623 (1.261–2.088)	< 0.001^a^		
Size, height	129	1.679 (1.317–2.141)	< 0.001^a^	0.671 (0.521–0.864)	0.002^a^
Tumour type, wall thickening	129				
Yes	66	1.107 (0.554–2.215)	0.774		
No	63	Reference			
Tumour type, polypoid	129				
Yes	35	Reference			
No	94	2.277 (1.002–5.171)	0.049^a^		
Tumour type, mass forming	129				
Yes	28	2.171 (0.923–5.107)	0.076		
No	101	Reference			
Involved wall, hepatic side	129				
Yes	9	2.528 (0.604–10.586)	0.204		
No	120	Reference			
Involved wall, peritoneal side	129				
Yes	5	0.284 (0.031–2.618)	0.267		
No	124	Reference			
Involved wall, both sides	129				
Yes	115	Reference			
No	14	1.212 (0.399–3.677)	0.735		
Liver invasion	129				
Yes	64	4.655 (2.208–9.810)	< 0.001^a^	0.369 (0.161–0.843)	0.018^a^
No	65	Reference			
Bile duct invasion	129				
Yes	24	1.005 (0.413–2.447)	0.992		
No	105	Reference			
Suspicion of LN (imaging)	129				
Yes	65	1.711 (0.850–3.443)	0.132		
No	64	Reference			
T stage (imaging)	129				
I	7	0.368 (0.041–3.276)	0.370		
II	61	Reference			
III	59	4.000 (1.871–8.553)	< 0.001^a^		
IV	2	2.211 (0.131–37.245)	0.582		
T1WI signal	129				
Iso- to hyperintense	61	Reference			
Hypointese	68	1.440 (0.717–2.892)	0.305		
T2WI signal	129				
Hyperintense	125	Reference			
Iso- to hypointense	4	1.193 (0.163–8.739)	0.862		
DWI signal	129				
Hyperintense	87	Reference			
Slightly hyperintense	42	0.543 (0.254–1.159)	0.114		
Arterial phase hyperenhancement	129				
Yes	64	Reference			
No	65	3.300 (1.598–6.814)	0.001^a^		
Arterial phase ring enhancement	129				
Yes	42	1.486 (0.709–3.115)	0.294		
No	87	Reference			
Arterial phase hypo-enhancement	129				
Yes	23	4.317 (1.574–11.840)	0.004^a^	0.331 (0.110–0.998)	0.0496^a^
No	106	Reference			
Delayed enhancement	129				
Yes	19	0.841 (0.314–2.252)	0.731		
No	110	Reference			
Wash in and wash out	129				
Yes	31	Reference			
No	98	0.869 (0.387–1.952)	0.734		
Continuous enhancement	129				
Yes	79	0.983 (0.483–2.001)	0.962		
No	50	Reference			
CER, AP	129	0.638 (0.470–0.866)	0.004^a^		
LLC, AP	129	0.295 (0.143–0.612)	0.001^a^		
LMC, AP	129	0.623 (0.389–0.996)	0.048^a^		
CER, PVP	129	0.593 (0.424–0.829)	0.002^a^		
LLC, PVP	129	0.139 (0.047–0.412)	< 0.001^a^		
LMC, PVP	129	0.821 (0.548–1.230)	0.339		
CER, DP	129	0.692 (0.504–0.951)	0.023^a^		
LLC, DP	129	0.166 (0.051–0.544)	0.003^a^		
LMC, DP	129	0.772 (0.536–1.112)	0.164		
Necrosis	129				
Yes	29	2.850 (1.201–6.762)	0.018^a^		
No	100	Reference			
Peripheral liver parenchyma enhancement	129				
Yes	72	Reference			
No	57	0.304 (0.145–0.634)	0.001^a^		

*TLSs* tertiary lymphoid structures, *CA199* carbohydrate antigen 199, *CEA* carcinoembryonic antigen, *LN* lymph nodes, *WI* weighted imaging, *DWI* diffusion weighted imaging, *AP* arterial phase, *PVP* portal vein phase, *DP* delayed phase, *CER* contrast enhancement ratio = (AP_mean_ − Mask_mean_)/Mask_mean_ or (PVP_mean_ – Mask_mean_)/Mask_mean_ or (DP_mean_ – Mask_mean_)/Mask_mean_, *LLC* lesion-to-liver contrast ratio = (AP_mean_ – AP_live_)/AP_liver_ or (PVP_mean_ – PVP_live_)/PVP_liver_ or (DP_mean_ – DP_live_)/DP_liver_, *LMC* lesion-to-muscle contrast ratio = (AP_mean_ – AP_muscle_)/AP_muscle_ or (PVP_mean_ – PVP_muscle_)/PVP_muscle_ or (DP_mean_ – DP_muscle_)/DP_muscle_

^a^ Data are statistically significant results

### Radiomics model

**Fig. 4:**
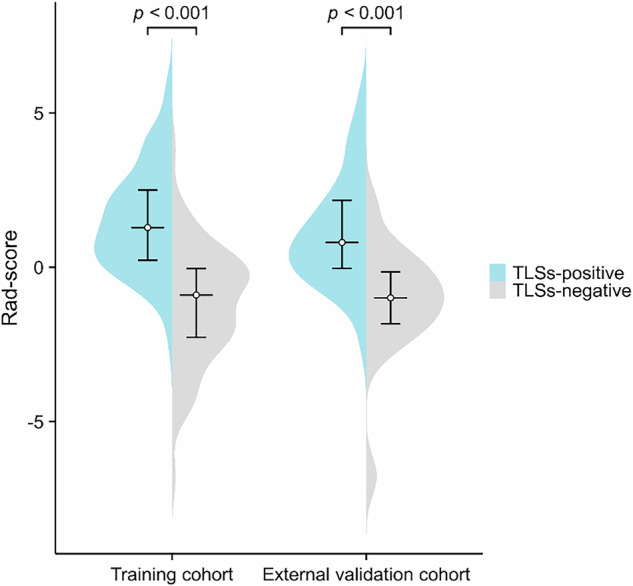
Violin plots comparing the Rad-score of the TLSs-positive and TLSs-negative groups in the training and external validation cohorts

Robust inter-observer agreement was observed for 629 features (interclass correlation coefficient range, 0.845–0.999; Supplementary Material 8: Table 5). Following the elimination of redundant features through variance, 316 features were retained. Further refinement via the discarding of irrelevant features through correlation reduced the count to 78 features, and multivariate logistic regression analysis reduced the count to eight features, forming the basis of the radiomics model (Rad-score). Heatmaps illustrating correlations between these features at various stages in model development are provided in Supplementary Material 9: Fig. 4. Coefficients and ORs for the Rad-score signatures are provided in Supplementary Material 10: Table 6. Rad-scores for patients with TLS-positive GBC were considerably higher than those with TLS-negative GBC in both the training and external validation populations (1.50 ± 1.67 vs –1.22 ± 1.90, *p* < 0.001 and 1.13 ± 1.75 vs –1.13 ± 1.88, *p* < 0.001, respectively; Fig. 4).

### Combined model and model performance

After the univariate and multivariate analyses, the four variables (tumour height; liver invasion; arterial phase hypo-enhancement and Rad-score) were integrated into the combined model (Supplementary Material 11: Table 7). Each patient was scored according to the formula: Combined-score = 0.916 × Rad-score − 0.140 × tumour height − 1.181 × liver invasion (yes = 1; no = 0) − 0.128 × arterial phase hypo-enhancement (yes = 1; no = 0). Per-patient Rad-scores and Combined-scores are presented in Supplementary Material 12: Fig. 5.

**Fig. 5:**
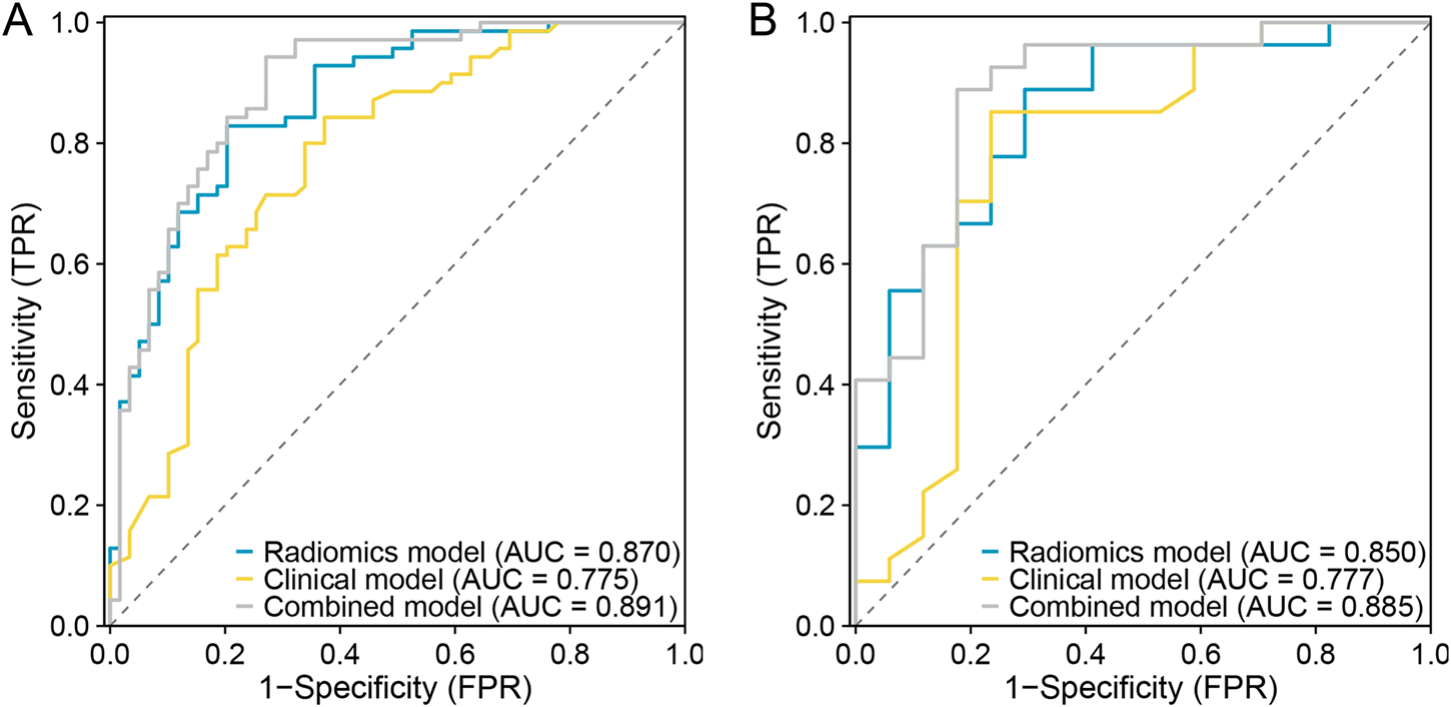
ROC curves of radiomics, clinical and combined models were compared in the training (**A**) and external validation (**B**) cohorts

Combined model outperformed the two separate models with data from the training cohort (AUC, 0.89 vs 0.87 and 0.76, respectively) and was found to be valid when tested with data from external validation cohort (Table 3 and Fig. 5). The DeLong test yielded *p* values of 0.02 for the radiomics vs clinical model, 0.18 for the radiomics model vs combined model and 0.0003 for the clinical model vs combined model for the training cohort, and 0.43, 0.33 and 0.10, respectively, for the external validation cohort.

**Table 3:** Performance comparison of the clinical, radiomics, and combined models in the training and external validation cohorts

Model	Training cohort (*n* = 129)	External validation cohort (*n* = 44)
Clinical model		
Sensitivity	0.843	0.851
Specificity	0.627	0.765
Accuracy	0.744	0.818
AUC (95% CI)	0.775 (0.693–0.858)	0.777 (0.614–0.939)
Radiomics model		
Sensitivity	0.829	0.889
Specificity	0.797	0.706
Accuracy	0.814	0.818
AUC (95% CI)	0.870 (0.809–0.931)	0.850 (0.731–0.968)
Combined model		
Sensitivity	0.943	0.889
Specificity	0.729	0.824
Accuracy	0.845	0.864
AUC (95% CI)	0.891 (0.833–0.949)	0.885 (0.777–0.992)

The results of combined model calibration and DCA were favourable for both cohorts (Hosmer–Lemeshow test, *p* = 0.24, 0.24 for the training, external validation cohorts; Supplementary Material 13: Fig. 6).

### Associations of combined-score estimates with survival in the training, external validation and immunotherapy cohorts

Sixty (46.5%) patients experienced recurrence during a median follow-up period of 36.1 months (IQR, 19.8–52.5 months). The median RFS was 10.8 months (IQR, 3.3–28.2 months).

**Fig. 6:**
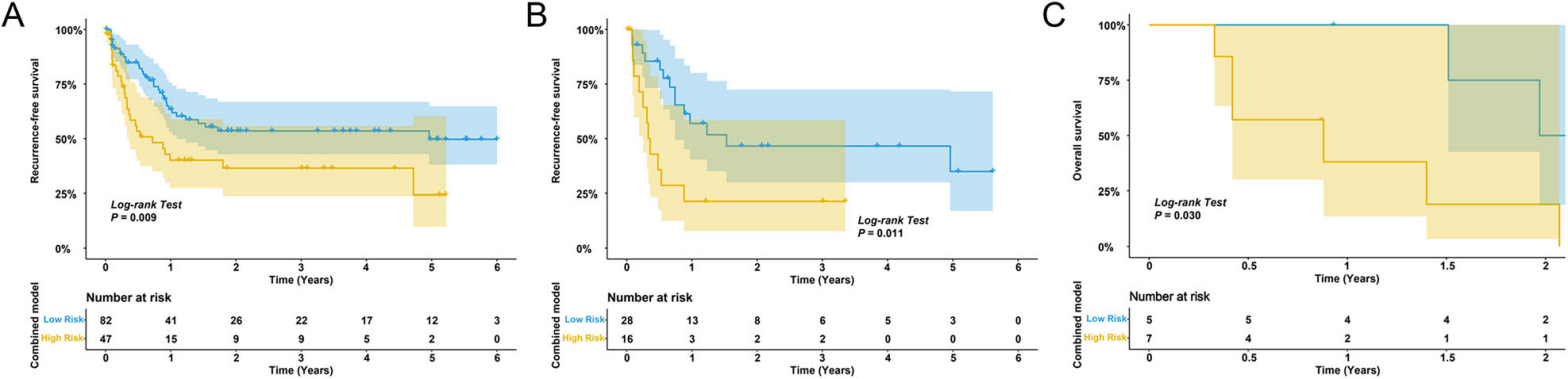
Kaplan–Meier curves for RFS of patients with GBC as categorised by the combined model (cut-off value = −0.474) in the training (**A**) and external validation cohorts (**B**). Kaplan–Meier curves for OS of patients with GBC as categorised by the combined model (cut-off value = −0.474) in the immunotherapy cohort (**C**)

In the training cohort, patients in the low-risk had a median RFS (Combined-score > −0.47) that was substantially longer than that of patients in the high-risk [59.6 months (95% CI: 13.0 months–NE) vs 8.7 months (95% CI: 4.4 months–NE; Fig. 6A]. The 6-, 12-, and 24-month RFS rates were 84.8%, 63.5% and 53.5% in the low-risk group and 53.4%, 40.1% and 36.5%, respectively, in the high-risk group. The median RFS of patients in the low-risk group was also significantly longer in the external validation cohort than that of patients in the high-risk group [18.4 months (95% CI: 8.9 months–NE) vs 4.2 months (95% CI: 3.0 months–NE); Fig. 6B]. The low-risk group’s median OS in the immunotherapy cohort was significantly longer than that of the high-risk group [24.7 months (95% CI: 18.1 months–NE) vs 10.5 months (95% CI: 5.1 months–NE); Fig. 6C].

## Discussion

This study identified the presence of intratumoural TLSs in patients with GBC as an independent predictor of RFS. This factor has been linked to better prognoses for patients with ICC [12, 20] and has been found to predict improved immunotherapeutic response for ICC and other solid tumours, such as hepatocellular carcinoma and melanoma, regardless of PD-L1 expression and CD8+ T-cell density [12–16]. In this study, three independent clinico-radiological predictors of TLS status (tumour height, liver invasion and arterial-phase hypo-enhancement) were included in the clinical model and eight features were included in the radiomics model. The final combined model integrated these two models and outperformed both when applied separately. Its survival stratification ability in surgical and immunotherapy cohorts was validated.

Choi et al [30] found that liver invasion was associated independently with resection margin positivity in patients with GBC. As per the system of the American Joint Committee on Cancer, gallbladder tumours classified as T3 have grown through the serosa and/or directly into the liver and/or a proximate extra-hepatic structure [31]. In a previous study, liver invasion was associated with the RFS of patients with completely resected GBC in a univariate analysis, but not in a multivariate analysis [32]. Consistent with the results of this study, a larger proportion of TLS-negative than TLS-positive cases showed liver invasion in the present study [33–35]. Whereas only two dimensions of tumour size have been considered in previous studies, we evaluated three dimensions and found that tumour height was an independent predictor of the presence of TLSs. On coronal images, tumour height was associated with the length of the adherent gallbladder wall, and thus likely the probability of wall invasion. The height of TLS-positive tumours was less than that of TLS-negative tumours, which may partly explain the more favourable prognoses of the former. Arterial enhancement has not been investigated in GBC, but it has been shown to be associated with ICC outcomes. Min et al [36] found that the ICC mortality and recurrence risks were lower for cases exhibiting diffuse arterial hyper-enhancement on MRI than for those showing peripheral rim enhancement or diffuse hypo-enhancement. We observed diffuse arterial hypo-enhancement in a larger proportion of TLS-negative than TLS-positive tumours, and this feature was associated with poorer prognosis. It is assumed that arterial hypo-enhancement in TLS-negative tumours might reflect less immune infiltration than the TLS-positive tumours, which is associated with a worse prognosis.

To date, no use of an MRI-based radiomics model for GBC survival prediction has been reported; however, CT-based radiomics models have been used for this purpose in several studies. Yin et al [37] established a radiomics model utilising portal venous phase CT images to discriminate benign and malignant gallbladder disease (AUC, 0.81); The model identified two shape features, two grey-level size zone (GLSZM) features, and one grey-level co-occurrence matrix (GLCM) feature as the top five predictive features. Similarly, the radiomics model features in this study were one shape feature, three GLSZM and GLCM features each, and one first-order feature. Meng et al [38] constructed a nomogram to predict the post–surgical resection survival of patients with GBC (AUC, 0.87); their radiomics model included two fissures and one GLSZM and GLCM feature each. Liu et al’s [39] radiomics model used three shape features and one first-order feature from the original images to predict lymph node metastases in GBC prior to surgery. In Gupta et al’s study, medium texture scale parameters, including both mean and kurtosis, or kurtosis alone, may help predict the histological grade and survival of GBC [40]. Thus, radiomics signatures may reflect the heterogeneity, microscopic pathological features, and immunophenotypes of TLS-positive and TLS-negative gallbladder tumours, enabling their differentiation.

In the training cohort, the specificity in the combined model was less than that in the radiomics model, while accuracy and sensitivity were higher. The possible reason may be as follows: The combined model integrates both clinical variables and radiomics features, offering a more comprehensive representation of the data. While this improves overall predictive performance (as reflected in higher accuracy and sensitivity), the inclusion of clinical variables may introduce additional variability, which could slightly lower the specificity. In contrast, the radiomics model is derived solely from imaging features, which may better capture tumour heterogeneity directly related to TLS status, thereby achieving higher specificity. As for the differences in sensitivity and specificity between the training and validation cohorts, it may be explained as follows: In the training cohort, the radiomics model demonstrates better specificity than the clinical model because it leverages high-dimensional features extracted from imaging data, which are optimised to fit the training data. However, in the external validation cohort, the radiomics model’s performance reflects its generalizability, resulting in higher sensitivity but a slight decrease in specificity compared to the training cohort.

### Limitations

This study was retrospective; the MRI dataset from the validation cohort was small, and it was inevitably affected by selection bias, as GBC is a relatively rare disease. However, the combined model was externally validated, demonstrating its reliability. Prospective multicentre studies with larger cohorts are needed. Second, manual segmentation was performed in this study; the reliability and reproducibility of the application of automatic segmentation for liver neoplasms should be explored. Third, the inclusion of clinical variables in the combined model may introduce additional variability, which could slightly lower the specificity. Therefore, the combined model has a limited effect on improving the performance of the radiomics model. Fourth, a difference in sample size in the training and external validation cohorts may cause greater statistical variability in the external validation cohort, affecting the sensitivity and specificity. As a result, a larger and more balanced sample size should be considered in the further study.

## Conclusion

The MRI-based radiomics combined model developed in this study may serve as a preoperative radiological tool for the detection of intratumoural TLS status. It outperformed independent radiomics and clinical models, with accurate prognostic stratification of the RFS of patients with GBC who had undergone surgery and the OS of patients with GBC who had undergone immunotherapy.

## Supplementary information

ELECTRONIC SUPPLEMENTARY MATERIAL

## Data Availability

The datasets used or analysed during the current study are available from the corresponding author on reasonable request. Requests to access these datasets could be directed to dr_fengye_ncc@163.com.

## Abbreviations

AUC: Area under the curve
CIs: Confidence intervals
CT: Computed tomography
DCA: Decision curve analysis
Fol–II: Secondary lymphoid follicles with germinal centres
GBC: Gallbladder cancer
GLCM: Grey level co-occurrence matrix
GLSZM: Grey level size zone matrix
ICC: Intrahepatic cholangiocarcinoma
IQRs: Interquartile ranges
MRI: Magnetic resonance imaging
ORs: Odds ratios
OS: Overall survival
PD-L1: Programmed death ligand-1
Rad-score: Radiomics score
RFS: Recurrence-free survival
ROCs: Receiver operating characteristic curves
TLSs: Tertiary lymphoid structures
VOI: Volume-of-interest
