# Supplementary material for: An MRI-based model for preoperative prediction of tertiary lymphoid structures in patients with gallbladder cancer
Source: Insights Imaging. 2025 Aug 30;16:189. doi: 10.1186/s13244-025-02007-4 (PMC12398454; doi:10.1186/s13244-025-02007-4)
Supplement: Supplementary file 1 — ELECTRONIC SUPPLEMENTARY MATERIAL [file 13244_2025_2007_MOESM1_ESM.pdf]

An MRI-based model for preoperative prediction of tertiary lymphoid structures in patients with gallbladder cancer

ELECTRONIC SUPPLEMENTARY MATERIAL

1. Supplementary material 1: MRI acquisition

Preoperative upper abdominal MRI examinations of all patients were performed using two scanners: GE Discovery MR750 3.0-T and GE SIGNA Pioneer 3-T (GE Healthcare, Boston, MA, USA). The MRI protocols included non-fat-suppressed coronal single-shot fast spin-echo (FSE) T2-weighted imaging (T2WI), axial fat-suppressed FSE T2WI, in-phase and out-of-phase T1-weighted imaging (T1WI), diffusion-weighted imaging (DWI), and contrast-enhanced-T1WI (CE-T1WI). DWI with an axial single-shot spin-echo echo-planar imaging sequence (DW gradients: b value 0/50 and 800 s/mm^2) was applied in three orthogonal directions (slice thickness/space: 5.0–8.0/1.0 mm, field-of-view: 36–42 cm, matrix size: 128×128, number of excitations: 4). For multi-phase CE-T1WI, unenhanced, early and late arterial phases (AP) (using the fluoro-triggering technique), portal venous phase (PVP) (60 s), and delayed phase (DP) (180 s) images were obtained using a three-dimensional T1WI breath-hold fat-suppressed spoiled gradient-recall echo Liver Acquisition with Volume Acquisition sequence before and after the intravenous administration of gadodiamide (0.5 mmol/mL, GE Healthcare) at a dose of 0.2 mL/kg and an injection rate of 2 mL/s [1]. Details of the sequences are shown in **Table 1**.

Table 1. The parameters details of the T1WI, T2WI, DWI and contrast enhanced-T1WI MRI sequences.

		GE Discovery MR750	GE SIGNA Pioneer
**Coronal FSE （SS-FSE）**	TR	1500 ms	1500 ms
	TE	87 ms	87 ms
	Pixel size	1.4 mm × 1.4 mm	1.4 mm × 1.4 mm
	Slice Thickness/space	4 mm/1 mm	4 mm/1 mm
	ETL	225	225
	Nex/Averages	1	1
	FOV	38-42 cm	38-42 cm
**Axial 3D T1WI Dual echo （LAVA Flex）**	TR	4.3 ms	4.3 ms
	TE	1.3/2.5 ms	1.3/2.5 ms
	Voxel size	1.6 mm × 1.9 mm ×4 mm	1.6 mm × 1.9 mm ×4 mm
	Nex/Averages	1	1
	FOV	36-42 cm	36-42 cm
**Axial FSE T2WI with fat supressed**	TR	Determined by respiratory rate	Determined by respiratory rate
	TE	85ms	85ms
	Pixel size	1.2 mm × 1.2 mm	1.2 mm × 1.2 mm
	Slice Thickness/space	5-8 mm/1 mm	5-8 mm/1 mm
	ETL	19	19
	Nex/Averages	2	2
	FOV	36-42 cm	36-42 cm

**Axial DWI**	B values	0/800 s/mm^2	0/800 s/mm^2
	TR	Determined by respiratory rate	Determined by respiratory rate
	TE	60 ms	60 ms
	Pixel size	3.0 mm × 2.2 mm	3.0 mm × 2.2 mm
	Thickness/space	5-8 mm/1 mm	5-8 mm/1 mm
	Nex/Averages	4	4
**Axial dynamic contrast-enhanced-T1WI （LAVA）**	The acquisition time of arterial phase	Using fluoro trigger technique	Using fluoro trigger technique
	The acquisition time of portal venous phase	60 s	60 s
	The acquisition time of delayed phase	180 s	180 s
	TR	3.7 ms	3.7 ms
	TE	1.5 ms	1.5 ms
	Voxel size	1.5 mm × 2.2 mm ×4-5 mm	1.5 mm × 2.2 mm ×4-5 mm
	Nex/Averages	1	1
	FOV	36-42cm	36-42cm

2. **Supplementary material 2: Table 2.** The MRI findings and signal characteristics.

MRI findings and signal characteristics	Definition[2-6]
Enhancement pattern	
Wash in and wash out	Arterial hyperenhancement with isointense or hypointense in the portal venous and delayed phase.
Centripetal enhancement	Rim or peripheral arterial-phase enhancement, and centripetal enhancement on the delayed phase.
Persistent enhancement	Hyperenhancement on the arterial phase and persistent enhancement in the portal venous and delayed phase.
Tumor size	
length	Measurement of the maximum diameter in the delayed phase on axial MRI.
width	Measurement of the maximum diameter perpendicular to the length measuring line in the delayed phase on axial MRI.
height	Measurement of the maximum diameter in the delayed phase on coronal MRI.
Tumor type	
wall thickening	Focal or diffuse wall thickening with heterogeneous enhancement of the GB.
polypoid	The polypoid tumor type was defined when a tumor protruded into the GB lumen and attached to the inner wall of GB with or without pedicle.
mass forming	The mass-forming tumor type was defined when a tumor mass replaced the GB fossa.
Liver invasion	Defined as a loss of fat plane between GBC and liver.
Bile duct invasion	Defined as that caused by either the main tumor or adjacent LN metastasis.
Suspicious lymph nodes (imaging)	Positive when a shortest diameter larger than 1 cm, central necrosis, or perinodal fat infiltration margin was present.
Intra-tumoral vessels	Blood vessels including hepatic arteries, hepatic veins, portal veins, and their branches go through the tumor.
Peripheral liver parenchyma enhancement	Gross hyperenhancement outside the tumor border in the arterial phase, becoming isointense in later dynamic phases compared with the background liver parenchyma.

Note: GB= gallbladder; GBC= gallbladder cancer; LN=lymph node.

3. Supplementary material 3: Quantification of enhancement values

We have attempted to perform the quantification of enhancement values as previously described[7; 8]: The lesions of pre-contrast (which named mask phase), arterial phase (AP), portal venous phase (PVP), delayed phase (DP), and adjacent hepatic parenchyma of AP, PVP, DP, muscle of AP, PVP, DP, excluding visible artifacts, were measured by manually delineating three ROIs at the three layers where the lesion was largest by Radiologist 1 under the supervision of senior Radiologist 2. And ROI of lesion was the largest circle along the inner margin of the lesion and the ROI of adjacent hepatic parenchyma or muscle was a circle of 1cm^2. The $Mask_{mean}$, AP_{mean}, AP_{liver}, AP_{muscle}, PVP_{mean}, PVP_{liver}, PVP_{muscle}, DP_{mean}, DP_{liver}, DP_{muscle}, were the mean signal intensity value of the three values. In the AP, the contrast enhancement ratio (CER_{AP}) was defined as $(AP_{mean} - Mask_{mean}) / Mask_{mean}$, the lesion-to-liver contrast ratio (LLC_{AP}) was defined as $(AP_{mean} - AP_{liver}) / AP_{liver}$, and the lesion-to-muscle contrast ratio (LMC_{AP}) was defined as $(AP_{mean} – AP_{muscle}) / AP_{muscle}$. In the PVP, the CER_{PVP} was defined as $(PVP_{mean} - Mask_{mean}) / Mask_{mean}$, the LLC_{PVP} was defined as $(PVP_{mean} - PVP_{liver}) / PVP_{liver}$, and the LMC_{PVP} was defined as $(PVP_{mean} – PVP_{muscle}) / PVP_{muscle}$. In the DP, the CER_{DP} was defined as $(DP_{mean} - Mask_{mean}) / Mask_{mean}$, the LLC_{DP} was defined as $(DP_{mean} - DP_{liver}) / DP_{liver}$, and the LMC_{DP} was defined as $(DP_{mean} – DP_{muscle}) / DP_{muscle}$.

4. **Supplementary material 4:** The examples of tumour segmentation for three tumor morphological types (wall thickening: figure 1; polypoid: figure 2; mass forming: figure 3).

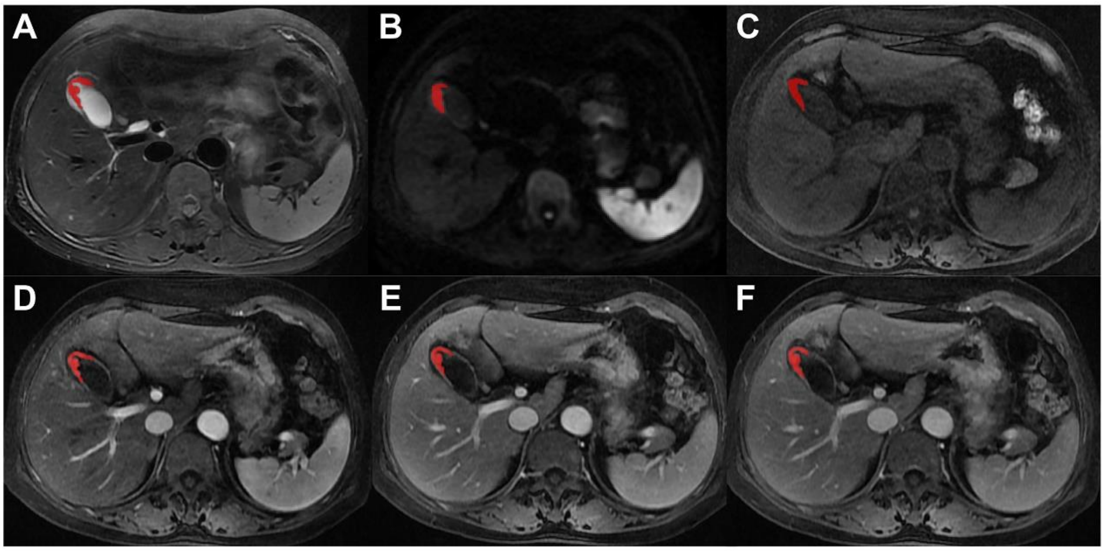

Figure 1: The tumour segmentation for wall thickening type were delineated manually using ITK-SNAP on axial T2-weighted (A), diffusion-weighted (B), non-enhanced T1-weighted (C), arterial phase (D), portal venous phase (E) and delayed phase (F) images.

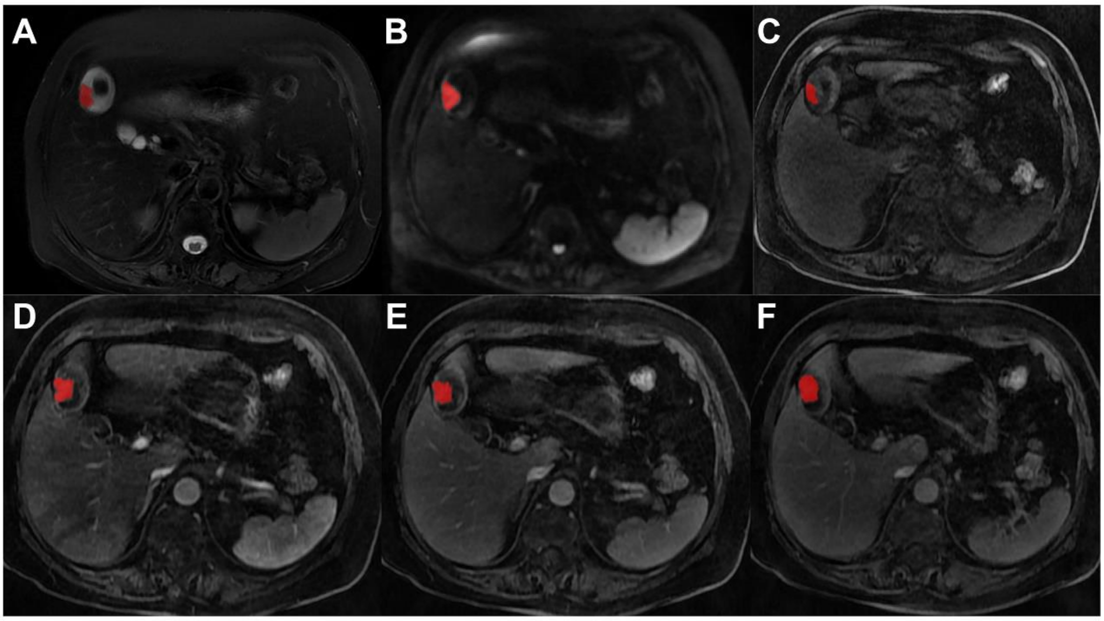

Figure 2: The tumour segmentation for polypoid type were delineated manually using ITK-SNAP on axial T2-weighted (A), diffusion-weighted (B), non-enhanced T1-weighted (C), arterial phase (D), portal venous phase (E) and delayed phase (F) images.

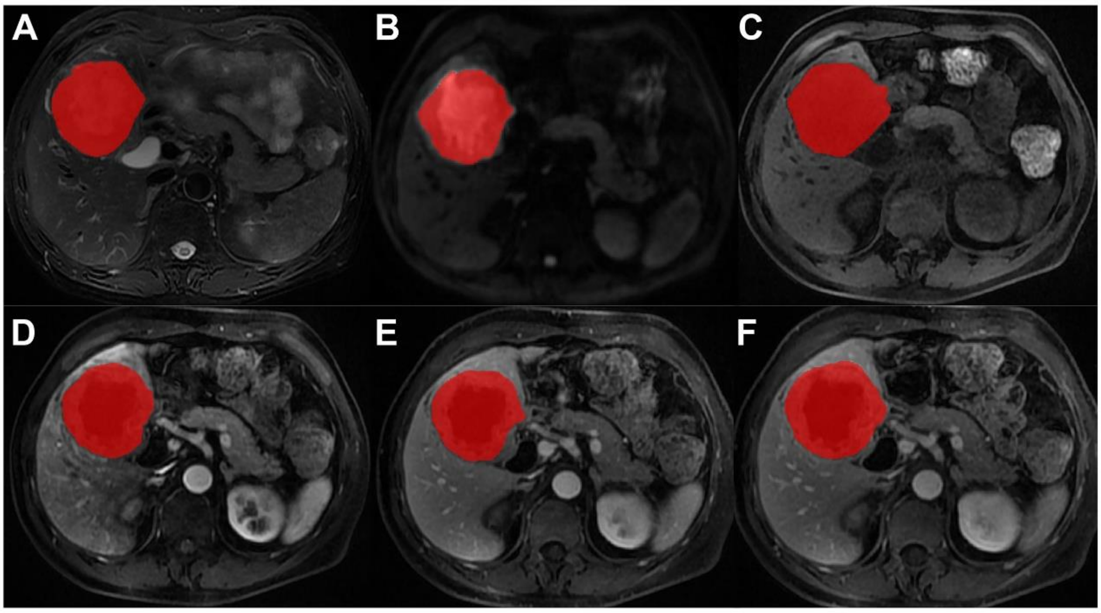

Figure 3: The tumour segmentation for mass forming type were delineated manually using ITK-SNAP on axial T2-weighted (A), diffusion-weighted (B), non-enhanced T1-weighted (C), arterial phase (D), portal venous phase (E) and delayed phase (F) images.

5. Supplementary material 5: The image pre-processing steps and process of radiomics.

The image pre-processing included two steps. First, the signal intensity distribution in the same tissue may be uneven, owing to the design and characteristics of the coil. Therefore, each MRI scan of each patient was normalized with Z-scores according to the following formula to obtain a standard normal distribution of image intensities: $\frac{s(x-\mu_x)}{\sigma_x}s$. Second, all the mask, T2WI, DWI, AP, PVP, DP images were resampled in all three dimensions via linear interpolation to ensure consistent voxel sizes (1×1×1 mm^3) and resolutions across all the sites and patients; subsequently, the radiomics features were extracted from each VOI using in-house software written in Python (pyradiomics version 2.12; https://pyradiomics.readthedocs. io/en/2.1.2/). A total of 14 shape features were extracted from the mask, T2WI, DWI, AP, PVP, and DP images. Subsequently, 93 radiomics features from each sequence were extracted, as follows: first-order (*n*=25), grey level co-occurrence matrix (GLCM) (*n*=22), grey level run length matrix (GLRLM) (*n*=16), grey level size zone matrix (GLSZM) (*n*=16), and grey level dependence matrix (GLDM) (*n*=14) features. In total, 642 features were extracted from 6 sequences for each patient: 6*(14 shape features + 25 first-order features + 22 GLCM features + 16 GLRLM features + 16 GLSZM features + 14 GLDM features).

A subset of regions of interest (ROIs) (30 patients) was redrawn by Radiologist 2 to calculate the reliability using the interclass correlation coefficient. Features with an interclass correlation coefficient exceeding 0.80 were retained. Three feature selection methods were used: variance, correlation, and multivariate logistic regression. First, a variance analysis was performed to eliminate features with minimal variability among patients, with a threshold of 1.0. The retained multi-VOI features were combined and selected using correlation, at a cutoff value of 0.7. Multivariate logistic regression was finally employed to select the most relevant features for predicting the status of TLS, at a threshold of 0.1, to derive the ultimate multi-sequence, multi-VOI radiomics model. The Rad-score was calculated using a linear combination of the selected features weighted by their respective logistic coefficients for each patient.

6. **Supplementary material 6: Table3. Univariate and multivariate cox regression analysis of clinical characteristics in patients with gallbladder cancer for RFS in the training cohort.**

Characteristics	Total(N)	Univariate analysis		Multivariate analysis	
		Hazard ratio (95% CI)	P value	Hazard ratio (95% CI)	P value
Intra-tumoral TLSs	129				
positive	70	Reference		Reference	
negative	59	2.236 (1.339 - 3.736)	**0.002***	1.863 (1.011 - 3.432)	**0.046***
Sex	129				
male	59	Reference			
female	70	0.696 (0.419 - 1.157)	0.162		
Age	129	1.011 (0.982 - 1.041)	0.470		
Cholecystitis	129				
yes	7	1.983 (0.785 - 5.010)	0.148		
no	122	Reference			
CA199>37U/ml	129				
yes	55	2.259 (1.350 - 3.781)	**0.002***	1.767 (0.997 - 3.133)	0.051
no	74	Reference		Reference	
CEA>5ng/mL	129				
yes	39	1.263 (0.736 - 2.166)	0.397		
no	90	Reference			
Location, neck	129				
yes	26	1.729 (0.971 - 3.078)	0.063		
no	103	Reference			
Location, body	129				
yes	27	0.572 (0.290 - 1.129)	0.107		
no	102	Reference			

Characteristics	Total(N)	Univariate analysis		Multivariate analysis	
		Hazard ratio (95% CI)	P value	Hazard ratio (95% CI)	P value
Location, fundus	129				
yes	51	Reference			
no	78	1.192 (0.705 - 2.016)	0.513		
Location, diffuse	129				
yes	25	1.332 (0.706 - 2.513)	0.376		
no	104	Reference			
Size, length	129	1.149 (1.019 - 1.294)	**0.023***	0.907 (0.633 - 1.299)	0.594
Size, width	129	1.233 (1.087 - 1.398)	**0.001***	1.329 (0.849 - 2.081)	0.213
Size, height	129	1.170 (1.050 - 1.302)	**0.004***	0.926 (0.693 - 1.237)	0.603
Tumor type, wall thickening	129				
yes	66	1.119 (0.673 - 1.860)	0.664		
no	63	Reference			
Tumor type, polypoid	129				
yes	35	Reference		Reference	
no	94	1.974 (1.025 - 3.801)	**0.042***	1.040 (0.504 - 2.145)	0.916
Tumor type, mass forming	129				
yes	28	1.706 (0.972 - 2.994)	0.063		
no	101	Reference			
Involved wall, hepatic side	129				
yes	9	0.183 (0.025 - 1.323)	0.093		
no	120	Reference			
Involved wall, peritoneal side	129				

Characteristics	Total(N)	Univariate analysis		Multivariate analysis	
		Hazard ratio (95% CI)	P value	Hazard ratio (95% CI)	P value
yes	5	0.000 (0.000 - Inf)	0.996		
no	124	Reference			
Involved wall, both sides	129				
yes	115	Reference		Reference	
no	14	0.115 (0.016 - 0.830)	**0.032***	0.188 (0.024 - 1.456)	0.110
Liver invasion	129				
yes	64	1.969 (1.174 - 3.305)	**0.010***	0.751 (0.295 - 1.914)	0.549
no	65	Reference		Reference	
Bile duct invasion	129				
yes	24	1.698 (0.930 - 3.100)	0.085		
no	105	Reference			
Regional LN (imaging)	129				
yes	65	1.378 (0.829 - 2.291)	0.216		
no	64	Reference			
Liver metastasis	129				
yes	6	2.277 (0.821 - 6.313)	0.114		
no	123	Reference			
T stage	129				
I	7	1.319 (0.390 - 4.453)	0.656	1.701 (0.491 - 5.894)	0.402
II	61	Reference		Reference	
III	59	2.613 (1.510 - 4.524)	**< 0.001***	2.032 (0.790 - 5.228)	0.141
IV	2	1.816 (0.242 - 13.614)	0.561	1.284 (0.139 - 11.860)	0.826
Gallstone	129				

Characteristics	Total(N)	Univariate analysis		Multivariate analysis	
		Hazard ratio (95% CI)	P value	Hazard ratio (95% CI)	P value
yes	25	1.186 (0.629 - 2.235)	0.598		
no	104	Reference			
Intrahepatic bile duct dilatation	129				
yes	54	Reference			
no	75	1.296 (0.766 - 2.192)	0.334		
Extrahepatic bile duct dilatation	129				
yes	58	Reference			
no	71	1.479 (0.874 - 2.502)	0.144		

Note: TLSs, tertiary lymphoid structures; CA199, carbohydrate antigen 199; CEA, carcinoembryonic antigen; LN, lymph nodes.

7. **Supplementary material 7: Table 4.** The interclass correlation coefficient of imaging semantics among radiologist 1 and radiologist 2.

Image semantics, n (%)	ICC	95% CI	*P* value
Location, neck	0.951	0.931-0.965	< 0.001
Location, body	0.909	0.874-0.935	< 0.001
Location, base	0.984	0.977-0.989	< 0.001
Location, diffuse	0.975	0.965-0.982	< 0.001
Tumor type, wall thickening	0.969	0.957-0.978	< 0.001
Tumor type, polypoid	0.941	0.918-0.958	< 0.001
Tumor type, mass forming	0.935	0.908-0.953	< 0.001
Involved wall, hepatic side	0.937	0.913-0.955	< 0.001
Involved wall, peritoneal side	0.886	0.842-0.918	< 0.001
Involved wall, both sides	0.920	0.889-0.943	< 0.001
Liver invasion	0.954	0.935-0.967	< 0.001
Bile duct invasion	0.923	0.892-0.945	< 0.001
Suspicious LN (imaging)	0.954	0.935-0.967	< 0.001
Liver metastasis	0.920	0.888-0.943	< 0.001
Intrahepatic bile duct dilatation	0.968	0.955-0.977	< 0.001
Extrahepatic bile duct dilatation	0.984	0.978-0.989	< 0.001
T1WI signal	0.969	0.957-0.978	< 0.001
T2WI signal	0.886	0.842-0.918	< 0.001
DWI signal	0.965	0.951-0.975	< 0.001
Arterial phase hyperenhancement	0.985	0.978-0.989	< 0.001
Arterial phase ring enhancement	0.948	0.927-0.963	< 0.001
Arterial phase hypoenhancement	0.947	0.926-0.963	< 0.001
Delayed enhancement	0.936	0.910-0.954	< 0.001
Wash in and wash out	0.979	0.970-0.985	< 0.001
Continuous enhancement	0.951	0.931-0.965	< 0.001
Necrosis	0.936	0.911-0.955	< 0.001
Intra-tumoral vessels	0.693	0.591-0.773	< 0.001

Peripheral liver parenchyma enhancement	0.953	0.934-0.967	< 0.001

Note: LN, lymph nodes; WI, weighted imaging; DWI, diffusion weighted imaging.

8. **Supplementary material 8: Table 5.** The interclass correlation coefficient of the extracted radiomics features from the ROIs of thirty GBC patients by two radiologists.

Radiomics features	T2WI	DWI	non-enhanced T1-weighted	AP	PVP	DP
original_shape_Elongation	0.967	0.95375	0.982316	0.982316	0.972989	0.972304
original_shape_Flatness	0.9752	0.96894	0.9912	0.9912	0.991629	0.981443
original_shape_LeastAxisLength	0.9947	0.99635	0.9986	0.9986	0.998512	0.998208
original_shape_MajorAxisLength	0.9983	0.99768	0.99848	0.99848	0.997599	0.998871
original_shape_Maximum2DDiameterColumn	0.9982	0.99756	0.997997	0.997997	0.997976	0.998941
original_shape_Maximum2DDiameterRow	0.9985	0.99879	0.99879	0.99879	0.997036	0.998413
original_shape_Maximum2DDiameterSlice	0.995	0.99633	0.998823	0.998823	0.997856	0.99801
original_shape_Maximum3DDiameter	0.9992	0.9994	0.998809	0.998809	0.996991	0.999131
original_shape_MeshVolume	0.9943	0.99327	0.998528	0.998528	0.998869	0.99794
original_shape_MinorAxisLength	0.994	0.99681	0.998222	0.998222	0.997787	0.99729
original_shape_Sphericity	0.9761	0.95834	0.983659	0.983659	0.97791	0.947545
original_shape_SurfaceArea	0.9971	0.99882	0.999127	0.999127	0.999335	0.998653
original_shape_SurfaceVolumeRatio	0.9793	0.97914	0.9843	0.9843	0.983119	0.97949
original_shape_VoxelVolume	0.9943	0.99336	0.998529	0.998529	0.99887	0.997938
original_firstorder_10Percentile	0.9882	0.95809	0.995156	0.97727	0.991013	0.987755
original_firstorder_90Percentile	0.9921	0.99861	0.998335	0.997516	0.997938	0.992439
original_firstorder_Energy	0.9913	0.99906	0.999658	0.998225	0.996677	0.993414
original_firstorder_Entropy	0.9846	0.99334	0.989702	0.993845	0.993452	0.989798
original_firstorder_InterquartileRange	0.9865	0.97408	0.988642	0.966423	0.994017	0.980797
original_firstorder_Kurtosis	0.783	0.98034	0.98389	0.967413	0.977295	0.991898
original_firstorder_Maximum	0.9541	1	0.998677	1	0.999885	0.997051
original_firstorder_MeanAbsoluteDeviation	0.9882	0.98641	0.992418	0.982425	0.996071	0.989479
original_firstorder_Mean	0.9941	0.99441	0.996977	0.99498	0.994852	0.989583
original_firstorder_Median	0.9934	0.99384	0.989391	0.995408	0.993053	0.984087
original_firstorder_Minimum	0.8613	0.88271	0.974725	0.91605	0.911112	0.884176
original_firstorder_Range	0.9385	0.99514	0.99756	0.994035	0.988099	0.977066
original_firstorder_RobustMeanAbsoluteDeviation	0.986	0.98097	0.99124	0.973227	0.996255	0.984847
original_firstorder_RootMeanSquared	0.9943	0.9963	0.997989	0.996168	0.995658	0.989726
original_firstorder_Skewness	0.8706	0.97109	0.992932	0.988701	0.970618	0.97095

original_firstorder_TotalEnergy	0.991	0.99845	0.999269	0.996615	0.996751	0.989244
original_firstorder_Uniformity	0.9891	0.99514	0.983869	0.993908	0.991857	0.989624
original_firstorder_Variance	0.9927	0.98765	0.992824	0.982813	0.995242	0.988102
original_firstorder_lz05Percentile	0.9768	0.94161	0.990828	0.96651	0.982407	0.97885
original_firstorder_lz10Percentile	0.9882	0.95809	0.995156	0.97727	0.991013	0.987755
original_firstorder_lz25Percentile	0.9943	0.9844	0.995479	0.988874	0.993646	0.991167
original_firstorder_lz50Percentile	0.9934	0.99384	0.989391	0.995408	0.993053	0.984087
original_firstorder_lz75Percentile	0.9926	0.99678	0.997765	0.996268	0.996126	0.986584
original_firstorder_lz90Percentile	0.9921	0.99861	0.998335	0.997516	0.997938	0.992439
original_firstorder_lz95Percentile	0.9933	0.99939	0.997331	0.998581	0.99896	0.997332
original_glcm_Autocorrelation	0.9383	0.97548	0.944268	0.981997	0.950243	0.89537
original_glcm_JointAverage	0.9245	0.97659	0.940183	0.984565	0.941744	0.89715
original_glcm_ClusterProminence	0.9991	0.99343	0.974657	0.982428	0.992399	0.985746
original_glcm_ClusterShade	0.9978	0.97837	0.984617	0.993843	0.992686	0.998336
original_glcm_ClusterTendency	0.9944	0.99019	0.994231	0.976105	0.992818	0.975734
original_glcm_Contrast	0.9896	0.98783	0.983251	0.994525	0.994824	0.99536
original_glcm_Correlation	0.9493	0.96983	0.968999	0.984804	0.991613	0.985588
original_glcm_DifferenceAverage	0.9882	0.98746	0.99405	0.995713	0.99489	0.992252
original_glcm_DifferenceEntropy	0.9848	0.99163	0.994623	0.997298	0.9967	0.995116
original_glcm_DifferenceVariance	0.9912	0.98968	0.973711	0.995491	0.994818	0.995096
original_glcm_JointEnergy	0.9905	0.99127	0.988038	0.994481	0.994979	0.988477
original_glcm_JointEntropy	0.9786	0.99267	0.992799	0.99559	0.994557	0.987144
original_glcm_Imc1	0.9469	0.98637	0.977304	0.99521	0.9941	0.994716
original_glcm_Imc2	0.9735	0.97495	0.966315	0.988525	0.990445	0.988172
original_glcm_Idm	0.993	0.98985	0.995664	0.996954	0.994916	0.9901
original_glcm_Idmn	0.8413	0.93727	0.98829	0.989575	0.960693	0.981183
original_glcm_Id	0.9925	0.98985	0.995827	0.997171	0.995412	0.991016
original_glcm_Idn	0.9109	0.96548	0.99375	0.995354	0.977584	0.983889
original_glcm_InverseVariance	0.9891	0.98662	0.996686	0.992822	0.987789	0.982861
original_glcm_MaximumProbability	0.9916	0.992	0.983761	0.996641	0.993303	0.981984
original_glcm_SumEntropy	0.9769	0.99157	0.988641	0.992626	0.994177	0.984544
original_glcm_SumSquares	0.9939	0.99029	0.994262	0.979359	0.993593	0.978805
original_glrlm_GrayLevelNonUniformity	0.9985	0.99091	0.99957	0.999333	0.999127	0.998642
original_glrlm_GrayLevelNonUniformityNormalized	0.9873	0.99519	0.98484	0.992979	0.991574	0.991763
original_glrlm_GrayLevelVariance	0.9927	0.98802	0.990338	0.984371	0.994305	0.990185
original_glrlm_HighGrayLevelRunEmphasis	0.9462	0.97986	0.954606	0.980587	0.945434	0.87487
original_glrlm_LongRunEmphasis	0.9829	0.99144	0.998706	0.998165	0.99911	0.99768
original_glrlm_LongRunHighGrayLevelEmphasis	0.9324	0.97739	0.975396	0.975853	0.930381	0.780106

original_glrlm_LongRunLowGrayLevelEmphasis	0.9622	0.96672	0.915448	0.996232	0.952954	0.694756
original_glrlm_LowGrayLevelRunEmphasis	0.9425	0.89509	0.882311	0.953942	0.874104	0.779005
original_glrlm_RunEntropy	0.9784	0.99353	0.990405	0.994774	0.995248	0.992473
original_glrlm_RunLengthNonUniformity	0.9942	0.99545	0.997992	0.99636	0.996521	0.996351
original_glrlm_RunLengthNonUniformityNormalized	0.9961	0.9879	0.995218	0.998203	0.998082	0.996037
original_glrlm_RunPercentage	0.9965	0.9901	0.997392	0.998973	0.998655	0.997702
original_glrlm_RunVariance	0.9906	0.99247	0.999176	0.997924	0.999196	0.99725
original_glrlm_ShortRunEmphasis	0.9925	0.98901	0.995844	0.998818	0.99819	0.997376
original_glrlm_ShortRunHighGrayLevelEmphasis	0.9549	0.98032	0.965978	0.982097	0.949907	0.903302
original_glrlm_ShortRunLowGrayLevelEmphasis	0.9378	0.87399	0.902212	0.945395	0.721986	0.849773
original_glszm_GrayLevelNonUniformity	0.9905	0.99364	0.99901	0.990357	0.991686	0.990072
original_glszm_GrayLevelNonUniformityNormalized	0.9549	0.99416	0.976938	0.988871	0.971822	0.991572
original_glszm_GrayLevelVariance	0.978	0.99269	0.985239	0.986419	0.976046	0.981089
original_glszm_HighGrayLevelZoneEmphasis	0.966	0.98507	0.980551	0.975752	0.955345	0.936919
original_glszm_LargeAreaEmphasis	0.9996	0.96983	0.999831	0.999581	0.998164	0.999251
original_glszm_LargeAreaHighGrayLevelEmphasis	0.9998	0.99377	0.994064	0.999825	0.99358	0.902727
original_glszm_LargeAreaLowGrayLevelEmphasis	0.9985	0.97625	0.997772	0.998579	0.998738	0.904801
original_glszm_LowGrayLevelZoneEmphasis	0.9433	0.73543	0.947504	0.966935	0.778564	0.845357
original_glszm_SizeZoneNonUniformity	0.9446	0.9838	0.998781	0.989215	0.982988	0.973719
original_glszm_SizeZoneNonUniformityNormalized	0.9136	0.93895	0.933626	0.959629	0.929432	0.916303
original_glszm_SmallAreaEmphasis	0.9024	0.89449	0.928881	0.954855	0.939605	0.919847
original_glszm_SmallAreaHighGrayLevelEmphasis	0.9697	0.9867	0.984404	0.976191	0.958648	0.918007
original_glszm_SmallAreaLowGrayLevelEmphasis	0.6433	0.7613	0.9622	0.9685	0.831554	0.903005
original_glszm_ZoneEntropy	0.9614	0.99363	0.989376	0.993635	0.988868	0.990106
original_glszm_ZonePercentage	0.9906	0.98924	0.99045	0.992516	0.985545	0.9893
original_glszm_ZoneVariance	0.9996	0.96919	0.999831	0.999592	0.998171	0.999252
original_gldm_DependenceEntropy	0.9836	0.99625	0.989395	0.992777	0.995053	0.993634

original_gldm_DependenceNonUniformity	0.9953	0.9943	0.999099	0.998684	0.998153	0.99772
original_gldm_DependenceNonUniformityNormalized	0.9932	0.98913	0.985899	0.993545	0.991629	0.982814
original_gldm_DependenceVariance	0.9972	0.98318	0.993766	0.998882	0.997453	0.993248
original_gldm_GrayLevelNonUniformity	0.9988	0.98983	0.999602	0.999853	0.999731	0.999291
original_gldm_GrayLevelVariance	0.9927	0.98762	0.992746	0.982882	0.995125	0.988192
original_gldm_HighGrayLevelEmphasis	0.9421	0.97944	0.947715	0.981862	0.947065	0.884562
original_gldm_LargeDependenceEmphasis	0.9971	0.98693	0.998254	0.999282	0.998802	0.997596
original_gldm_LargeDependenceHighGrayLevelEmphasis	0.9399	0.97905	0.953424	0.981508	0.96359	0.858627
original_gldm_LargeDependenceLowGrayLevelEmphasis	0.9739	0.99616	0.909902	0.996006	0.970143	0.720269
original_gldm_LowGrayLevelEmphasis	0.9385	0.91395	0.866785	0.962963	0.92011	0.732407
original_gldm_SmallDependenceEmphasis	0.9876	0.98512	0.992079	0.993621	0.993724	0.992782
original_gldm_SmallDependenceHighGrayLevelEmphasis	0.9888	0.98444	0.987555	0.981829	0.964783	0.964454
original_gldm_SmallDependenceLowGrayLevelEmphasis	0.8828	0.73089	0.989477	0.975638	0.664786	0.954519

9. **Supplementary material 9: Figure 4.** The heatmaps of correlations between the features in the variance (A, D), correlation (B, E) and logistic regression (C, F) selection steps in the training and external validation cohort.

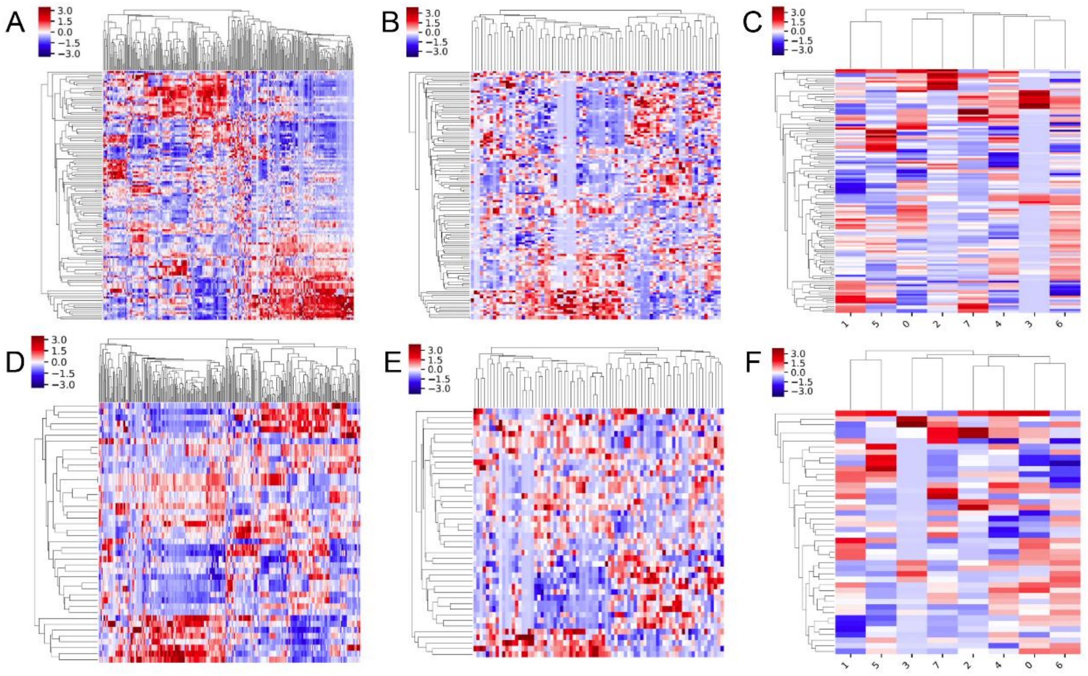

10. **Supplementary material 10: Table 6. The eight selected radiomics features to derive the Rad-score in the training cohort.**

Radiomics features	Coefficient	OR	95%CI	P value
MASK_original_firstorder_lz10Percentile	-1.1866	0.305	0.164-0.568	<0.001
AP_original_glszm_SmallAreaEmphasis	1.0235	2.783	1.559-4.967	0.001
T2_original_glcm_ClusterShade	0.4049	1.499	0.926-2.427	0.099
AP_original_glszm_LargeAreaLowGrayLevelEmphasis	-1.0366	0.355	0.164-0.766	0.008
DWI_original_shape_Sphericity	0.6740	1.962	1.203-3.199	0.007
MASK_original_glszm_SmallAreaLowGrayLevelEmphasis	-0.8706	0.419	0.23-0.761	0.004
T2_original_glcm_Idmn	-0.9332	0.393	0.206-0.751	0.005
PVP_original_glcm_ClusterProminence	-0.7517	0.472	0.257-0.864	0.015

11. Supplementary material 11: Table 7. Univariate and multivariate logistic regression analysis of clinical characteristics and Rad-score in patients with gallbladder cancer for TLSs in the training cohort.

Characteristics	Total(N)	Univariate analysis		Multivariate analysis	
		Odds Ratio (95% CI)	P value	Odds Ratio (95% CI)	P value
Rad-score	129	2.718 (1.888 – 3.915)	**< 0.001***	2.499 (1.663 – 3.755)	**< 0.001***
Size, height	129	0.595 (0.467 – 0.759)	**< 0.001***	0.869 (0.638 – 1.183)	0.373
Liver invasion	129				
yes	64	0.215 (0.102 – 0.453)	**< 0.001***	0.307 (0.112 – 0.841)	**0.022***
no	65	Reference		Reference	
Arterial phase hypo-enhancement	129				
yes	23	0.232 (0.084 – 0.635)	**0.004***	0.880 (0.239 – 3.234)	0.847
no	106	Reference		Reference	

*Data are statistically significant result.

12. Supplementary material 12: Figure 5. The Rad-score (A, B) and Combined-score (C, D) of each patient in training and external validation cohorts.

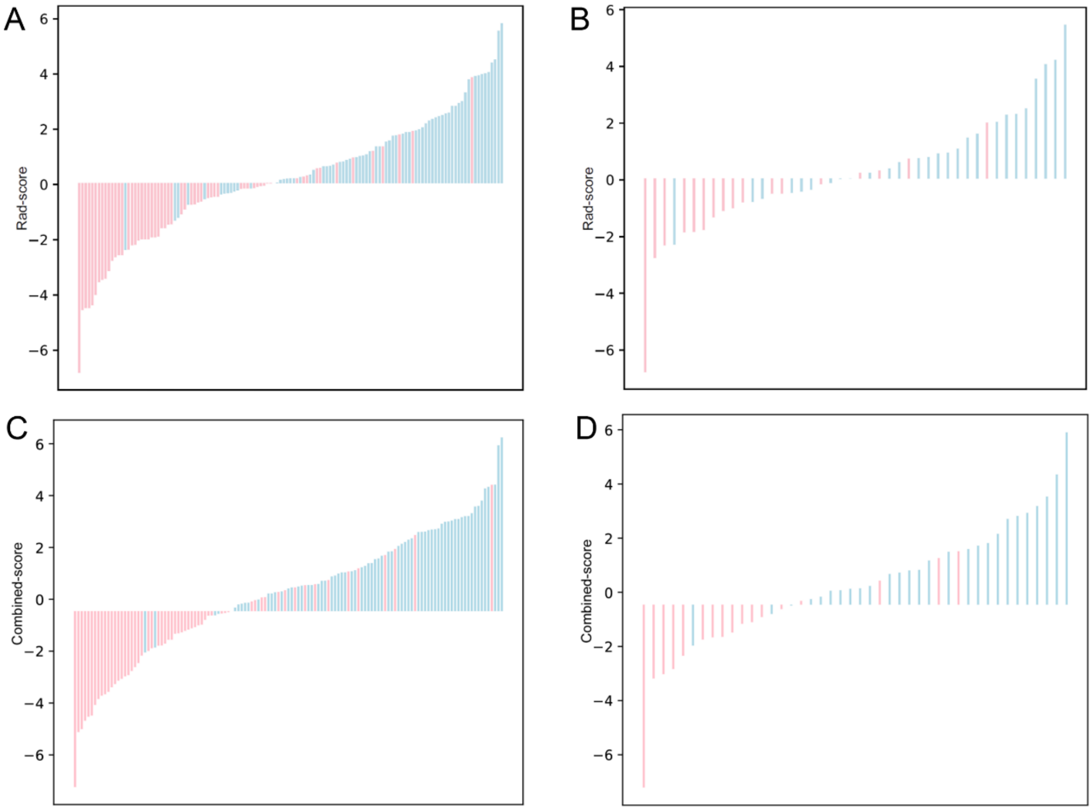

13. **Supplementary material 13: Figure 6.** Calibrations of the combined model in the training (A), external validation (B) cohorts. Decision curve analysis curves for the combined model in the training (C) and external validation (D) cohorts. The y-axis indicates the net benefit and x-axis indicates threshold probability. The red line represents net benefit of the combined model.

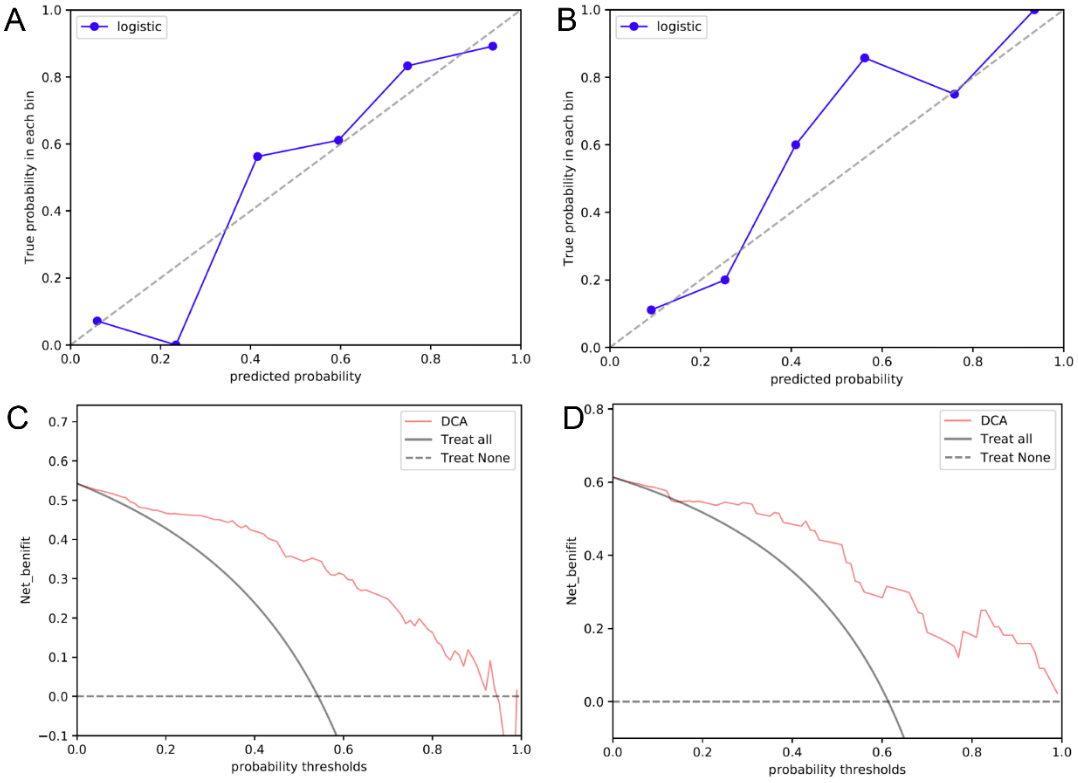

1 Donato H, França M, Candelária I, Caseiro-Alves F (2017) Liver MRI: From basic protocol to advanced techniques. European Journal of Radiology 93:30-39
2 Kim SJ, Lee JM, Lee ES, Han JK, Choi BI (2015) Preoperative staging of gallbladder carcinoma using biliary MR imaging. Journal of Magnetic Resonance Imaging : JMRI 41:314-321
3 Choi S-Y, Kim JH, Lim S, Lee JE, Park HJ, Lee B (2021) CT-based nomogram for predicting survival after R0 resection in patients with gallbladder cancer: a retrospective multicenter analysis. European Radiology 31:3336-3346
4 Min JH, Kang TW, Cha DI et al (2019) Apparent diffusion coefficient as a potential marker for tumour differentiation, staging and long-term clinical outcomes in gallbladder cancer.European Radiology 29:411-421
5 Min JH, Kim YK, Choi S-Y et al (2019) Intrahepatic Mass-forming Cholangiocarcinoma: Arterial Enhancement Patterns at MRI and Prognosis. Radiology 290:691-699
6 Yang Y, Zou X, Zhou W et al (2022) Multiparametric MRI-Based Radiomic Signature for Preoperative Evaluation of Overall Survival in Intrahepatic Cholangiocarcinoma After Partial Hepatectomy. Journal of Magnetic Resonance Imaging : JMRI 56:739-751
7 Fronda M, Doriguzzi Breatta A, Gatti M et al (2021) Quantitative assessment of HCC wash-out on CT is a predictor of early complete response to TACE. Eur Radiol 31:6578-6588
8 Gatti M, Calandri M, Bergamasco L et al (2020) Characterization of the arterial enhancement pattern of focal liver lesions by multiple arterial phase magnetic resonance imaging: comparison between hepatocellular carcinoma and focal nodular hyperplasia. Radiol Med 125:348-355
